# Far-infrared transparent conductors

**DOI:** 10.1038/s41377-023-01139-w

**Published:** 2023-04-21

**Authors:** Chaoquan Hu, Zijian Zhou, Xiaoyu Zhang, Kaiyu Guo, Can Cui, Yuankai Li, Zhiqing Gu, Wei Zhang, Liang Shen, Jiaqi Zhu

**Affiliations:** 1 State Key Laboratory of Superhard Materials, Key Laboratory of Automobile Materials of Ministry of Education, School of Materials Science and Engineering, Jilin Provincial International Cooperation Key Laboratory of High-Efficiency Clean Energy Materials, Jilin University, Changchun, 130012 China; 2 College of Information Science and Engineering, Jiaxing University, Jiaxing, 314001 China; 3 Electron Microscopy Center, Jilin University, Changchun, 130012 China; 4 State Key Laboratory of Integrated Optoelectronics, College of Electronic Science and Engineering, International Center of Future Science, Jilin University, Changchun, 130012 China; 5 National Key Laboratory of Science and Technology on Advanced Composites in Special Environments, Harbin Institute of Technology, Harbin, 150080 China

**Keywords:** Electronics, photonics and device physics, Optical materials and structures

## Abstract

The long-standing challenge in designing far-infrared transparent conductors (FIRTC) is the combination of high plasma absorption edge (*λ*_*p*_) and high conductivity (*σ*). These competing requirements are commonly met by tuning carrier concentration or/and effective carrier mass in a metal oxide/oxonate with low optical dielectric constant (*ε*_*opt*_ = 2–7). However, despite the high *σ*, the transparent band is limited to mid-infrared (*λ*_*p*_ < 5 μm). In this paper, we break the trade-off between high *σ* and *λ*_*p*_ by increasing the “so-called constant” *ε*_*opt*_ that has been neglected, and successfully develop the material family of FIRTC with *ε*_*opt*_ > 15 and *λ*_*p*_ > 15 μm. These FIRTC crystals are mainly octahedrally-coordinated heavy-metal chalcogenides and their solid solutions with shallow-level defects. Their high *ε*_*opt*_ relies on the formation of electron-deficiency multicenter bonds resulting in the great electron-polarization effect. The new FIRTC enables us to develop the first “continuous film” type far-infrared electromagnetic shielder that is unattainable using traditional materials. Therefore, this study may inaugurate a new era in far-infrared optoelectronics.

## Abstract (web summary)

The first family of far-infrared transparent conductive materials was developed by increasing the optical dielectric constant.

## Graphical abstract

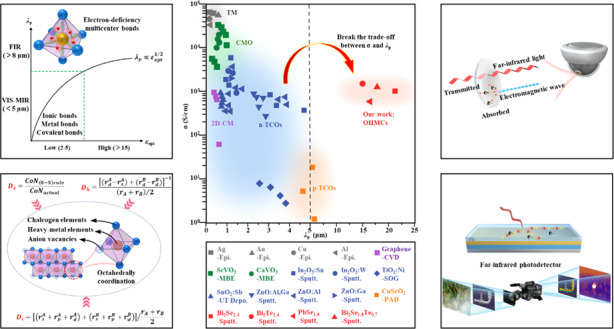

## Introduction

Transparent conductor (TC) refers to materials with high transmittance and high conductivity in the interesting band^1–3^. They are widely used as key materials in optoelectronic devices, including flat-panel displays^4^, solar cells^5,6^, and emerging flexible^7,8^ and transparent electronics^9–12^. Although TC in the visible, near-infrared, and mid-infrared bands have been developed, TC in the far-infrared band have not been designed. This greatly limits the development of far-infrared electromagnetic shielding^13,14^, infrared thermal camouflage^15^, photo detection^16–19^, biosensing^20^ technologies, and other fields. Therefore, we focus on far-infrared transparent conductors.

**Fig. 1:**
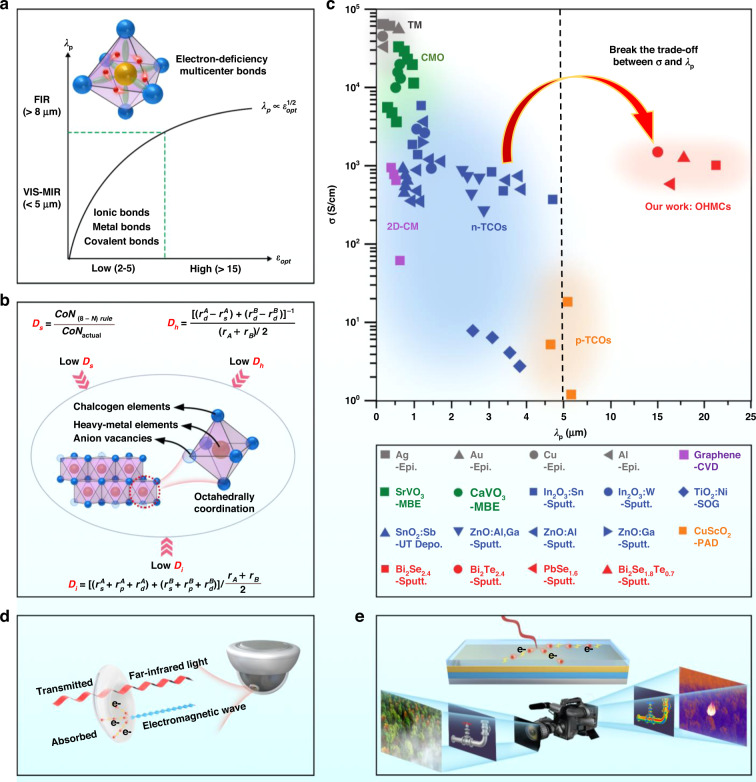
Material design of far-infrared transparency and conductivity. **a** The positive correlation between *λ*_*p*_ and *ε*_*opt*_. Typical materials with ionic, metal, and covalent bonds usually have smaller *ε*_*opt*_ and *λ*_*p*_, while materials with electron-deficiency multicenter bonds have greater *ε*_*opt*_ and *λ*_*p*_. **b** The three microscopic principles for the formation of the electron-deficiency multicenter bond, and the octahedrally-coordinated heavy-metal chalcogenides and their solid solutions selected by the three principles. The introduction of anion vacancies is to obtain shallow energy level defects and good conductivity. **c** The plasma absorption edge and room temperature conductivity of traditional transparent conductive materials and the four heavy-metal chalcogenides (red) that we prepared (Supplementary refs. 25–32), where gray, green, blue, orange, purple, and red represent thin metals (TM), correlated metal oxonates (CMO), n-type transparent conductive oxide (n-TCOs), p-type transparent conductive oxide (p-TCOs), 2D carbon materials (2D-CM), and octahedrally-coordinated heavy-metal chalcogenides (OHMCs), respectively. The materials and preparation methods are detailed in SI. The “continuous film” type far-infrared electromagnetic shielder (**d**) and far-infrared photodetector (**e**) based on far-infrared transparent conductors

Transmission is a phenomenon that incident light first refracts and then passes through a medium, which is mainly affected by three absorptions of intraband transition, interband transition, and lattice vibration. The ideal condition for far-infrared transparency is that the material has extremely low light absorption in the 8–12 μm. This requires that the interband transition wavelength of the material is less than 8 μm, lattice vibration and the plasma absorption edge (*λ*_*p*_) is greater than 12 μm^21^. According to Eq. 1, the *λ*_*p*_ depends on the speed of light *C*_0_ and screened plasma energy (*ω*_p_*). *ω*_p_* includes both the contribution of the ion lattice background and free electron gas to the polarization response. The value of *ω*_p_* depends on the optical dielectric constant *ε*_*opt*_, the effective carrier mass m*, the carrier concentration *n*, the vacuum dielectric constant *ε*_0_, and the unit charge *e*^21^. According to Eq. 2, the room-temperature conductivity *σ* depends on m*, *n*, relaxation time *τ*, and *e*. From the two Equations, *λ*_*p*_ is proportional to m* and inversely proportional to *n*, while *σ* is inversely proportional to m* and directly proportional to *n*. Therefore, there is a trade-off between high *λ*_*p*_ and high *σ* (Fig. 1). This has proved to be a classic problem that limits the development of physics, materials, and devices of far-infrared electronics^1^.

1 $$\lambda _P = \frac{{2\pi C_0}}{{\omega _p \ast }} = 2\pi C_0\sqrt {\frac{{m^ \ast \varepsilon _0\varepsilon _{opt}}}{{ne^2}}} \propto \frac{{m^ \ast \varepsilon _{opt}}}{n}$$

2 $$\sigma = \frac{{n{{{\mathrm{e}}}}^2\tau }}{{m^ \ast }} \propto \frac{{n\tau }}{{m^ \ast }}$$

Traditional transparent conductive film materials are mainly classified into the following five categories according to design strategy and chemical composition. Their *σ*, *n*, *τ*, m*, *ε*_*opt*_, and *λ*_*p*_ are summarized in Supplementary Information. (1) n-type transparent conductive oxide (n-TCOs)^22^. To obtain high *σ*, wide band gap binary oxides are used as the host, such as In_2_O_3_, ZnO, SnO_2_, and TiO_2_, and the doping concentration is maximized to ensure the electron concentration (Eq. 2). However, high electron concentration will inevitably reduce *λ*_*p*_ according to Eq. 1, which further decreases the infrared transmission properties (Fig. 1). (2) p-type transparent conductive oxides (p-TCOs)^23^. p-TCOs mainly includes Cu^+^-based delafossites, layered oxychalcogenides, *nd*^6^ spinel oxides, Cr^3+^-based oxides (3*d*^3^), post-transition metal oxides with lone pair state (*ns*^2^) and some Cu^+^-based halides and phosphides. The high local nature of O2*p*-derived valence band leads to a greater effective hole mass and low mobility. This results in that p-TCOs has a relatively large *λ*_*p*_, but a low *σ* (Fig. 1). (3) Correlated metal oxonates^21^. In materials such as SrVO_3_ and CaVO_3_, m* increases due to electron-electron interactions. It is possible to increase *λ*_*p*_ and maintain high *n* to obtain transparent conductive properties. However, *λ*_*p*_ is still too small because of the limited adjustment range of *n* and m* of these materials (Fig. 1). (4) Thin metals (nickel, silver, copper, etc.)^24^, and 2D carbon materials (graphene, carbon nanotubes)^25^. Many studies have shown that the electrical conductivity of these materials is proportional to their thickness, while the transmittance is inversely proportional to the thickness. Therefore, it is difficult to achieve a compromise between high electrical conductivity and infrared transmittance. The thin metals and 2D carbon materials generally have high electrical conductivity and poor infrared transmittance (Fig. 1). (5) Transparent conductive polymer. At present, transparent conductive polymers such as PANI:CSA and PEDOT:PSS have been used in flexible OLED devices in the visible to near-infrared band. However, these materials are not suitable for applications in the far-infrared band because of the presence of infrared absorption peaks caused by polar bonds^26^. In summary, researchers have studied the preparation, structure, and performance of various transparent conductive films and have made many important progresses. However, in these studies, researchers mainly focused on visible transparent conduction, and less attention were paid to infrared transparent conduction. Among all the materials that have been reported, the ITO film has the highest *λ*_*p*_ and *σ* compatibility. However, it can only achieve transparency within the mid-infrared band (*λ*_*p*_ < 5 μm) under the high conductivity (*σ* > 1000 S/cm). The trade-off between high *λ*_*p*_ and high *σ* are still not solved well (Fig. 1).

The *ε*_*opt*_ refers to the dielectric constant produced by electron displacement polarization in the ultraviolet-visible-near-infrared optical frequency range^27^. According to Eq. (1), a greater value of *ε*_*opt*_ can weaken the coulomb potential of ion core to free electrons, thereby increasing *λ*_*p*_. However, researchers have mainly focused on the effect of *n* and m* on *λ*_*p*_ and *σ*^1^, ignoring the effect of the “so-called constant” *ε*_*opt*_ on increasing *λ*_*p*_. The selected materials are mainly metal oxides/oxonates with small *ε*_*opt*_ (2–7). Although these materials have good electrical conductivity, the long-wave cutoff wavelength of transparent area is limited to the mid-infrared band (*λ*_*p*_ < 5 μm). In addition, studies have reported the effect of the doping^28^ and heating^29^ on increasing the *ε*_*opt*_ of the dielectric material. However, the effect of structure parameters such as the coordination number on *ε*_*opt*_ has not been well explored. It is still not clear how to design high-*ε*_*opt*_ materials at the atomic level.

This paper proposes a rational design strategy that realizes the synergy of far-infrared transparency and conductivity by increasing *ε*_*opt*_ for the first time, as shown in Fig. 1. In order to design high-*ε*_*opt*_ materials, we analyze the interesting phenomenon that the *ε*_*opt*_ (29.6) of crystalline Bi_2_Se_2.4_ is greater than that of amorphous Bi_2_Se_2.4_ (9.5). This shows that the electron-deficiency multicenter bond (EDMD) formed under high coordination number are an effective way to obtain high *ε*_*opt*_ (Fig. 1a). Because EDMD increases the Bohr radius and electron delocalization, resulting in a stronger electron-polarization effect than typical ionic, metal and covalent bonds (details in SI). To predict high-*ε*_*opt*_ materials, we prove that low ionization, low hybridization, and low saturation are the three necessary principles for the formation of electron-deficiency multicenter bond (Fig. 1b). Then, we sketch the map of high-*ε*_*opt*_ materials, and discover that high-*ε*_*opt*_ materials are mainly octahedral-coordinated heavy-metal chalcogenides and their solid solutions (Fig. 1b). To obtain good electrical conductivity under the premise of far-infrared transparency, we introduce shallow energy level defects such as anion vacancies in these high-*ε*_*opt*_ crystals. This realizes the integration of far-infrared transparency and conductivity (Fig. 1c).

To verify the concept, we prepared polycrystalline octahedrally-coordinated heavy-metal chalcogenides rich in anion vacancies with a thickness of 20–160 nm: r-Bi_2_Se_2.4_, r-Bi_2_Te_2.4_, r-Bi_2_Se_1.8_Te_0.7_ solid solution and c-PbSe_1.6_. The results show that these materials have the characteristics of electron-deficiency multicenter bond and high *ε*_*opt*_ (*ε*_*opt*_ > 15). Figure 1c shows the *λ*_*p*_ versus *σ* of these four high-*ε*_*opt*_ chalcogenides and traditional transparent conducting materials. The four high-*ε*_*opt*_ chalcogenides have high *σ* (600–2000 S/cm) and also have much greater *λ*_*p*_ (>15 μm) than that of traditional transparent conductive materials. Therefore, this new material family can be regarded as the first type of far-infrared transparent conductors (FIRTC). In addition, we develop the first “continuous film” type far-infrared electromagnetic shielder using r-Bi_2_Se_2.4_ (Fig. 1d), which have excellent performances that cannot be achieved by traditional materials. We expect that this FIRTC can also be applied to other technical fields that are beyond the reach of traditional TCs, such as far-infrared detection (Fig. 1e). This will lead to more new research into optoelectronic physics, materials, and devices.

## Results

### What is the microscopic origin of high *ε*_*opt*_?

**Fig. 2:**
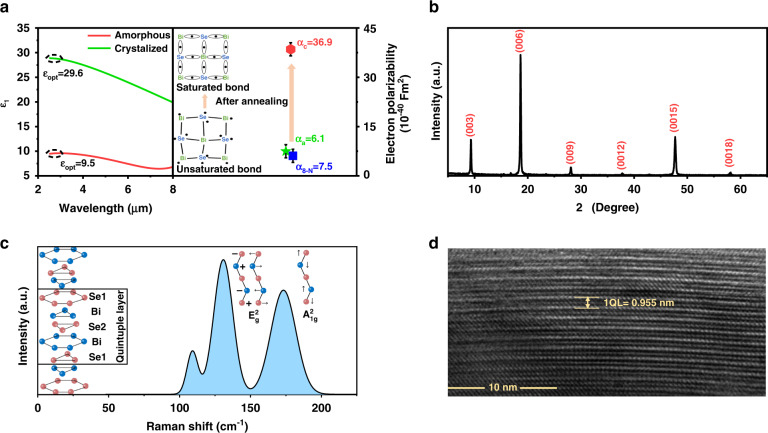
Optical dielectric constant and crystal structure of Bi_2_Se_2.4_ thin film. **a** The increase in *ε*_*opt*_ and *α*_*e*_ caused by the transition from amorphous to crystalline, where the inset is a schematic diagram of the transformation of saturated bonds to unsaturated bonds. **b** The XRD spectrum, **c** Raman spectrum, and **d** HRTEM image of crystalline Bi_2_Se_2.4_ thin film

Figure 2a plots the real part of the dielectric function of amorphous and crystalline Bi_2_Se_2.4_, where the real part of the dielectric function within 2.5–3.0 μm is approximately constant. Therefore, the average value of the dielectric constant in this band is taken as *ε*_*opt*_. This method of determining *ε*_*opt*_ has been reported in other studies^30^. The *ε*_*opt*_ of amorphous Bi_2_Se_2.4_ is 9.5. However, it is drastically increased to 29.6 after crystallization, an increase of 2 times. This indicates that the transition from amorphous to crystalline can significantly affect *ε*_*opt*_. The dramatic increase in *ε*_*opt*_ caused by this crystallization provides us a key to reveal the microscopic origin of high *ε*_*opt*_.

To reveal the microscopic origin of high *ε*_*opt*_, we used the Clausius-Mossotti equation^30^ $\frac{{\varepsilon _{{{{\mathrm{opt}}}}} - 1}}{{\varepsilon _{{{{\mathrm{opt}}}}} + 2}} = \frac{{N\alpha _{{{\mathrm{e}}}}}}{{3\varepsilon _0}}$ to calculate the electron polarizability α_e_ of the amorphous and crystalline Bi_2_Se_2.4_, as shown in Fig. 2a. As Bi_2_Se_2.4_ transformed from amorphous to crystalline, its electronic polarizability is drastically increased from 6.1 to 36.9 (details in SI). This indicates the appearance of the strong electron-polarization effect. To study the origin of the strong electronic polarization, we used the asymmetric diatomic molecular model^31^ and the atomic polarizabilities of Bi and Se to calculate the polarizability of bismuth selenide under the 8-N rule (details in SI). The results show that the electronic polarizability of amorphous Bi_2_Se_2.4_ (6.1) is very close to that of bismuth selenide (7.5) under the 8-N rule. This shows that the bonding characteristics of amorphous Bi_2_Se_2.4_ conform to the 8-N rule, and its chemical bond is saturated. However, the electronic polarizability of crystalline Bi_2_Se_2.4_ (36.9) is much higher than that of bismuth selenide under the 8-N rule (7.5). This shows that another unsaturated chemical bond forms in the crystalline Bi_2_Se_2.4_.

To identify the bonding structure of crystalline Bi_2_Se_2.4_, we conducted a series of experiments and theoretical calculations. In an X-ray diffraction spectrum (Fig. 2b), the appearance of (003), (006), (009), (0012), (0015), and (0018) diffraction peaks indicates that Bi_2_Se_2.4_ has a rhombohedral structure and good c-axis orientation. In a Raman spectrum (Fig. 2c), the appearance of strong peaks of E^2^_g_ and A^1^_2 g_ indicates that Bi_2_Se_2.4_ has a good layered structure^32^. In a HRTEM image (Fig. 2d), the film exhibits layered growth and the thickness of each QL layer is 0.955 nm. Therefore, the results indicate that the prepared Bi_2_Se_2.4_ thin film is a polycrystalline rhombohedral phase, abbreviated as r-Bi_2_Se_2.4_.

**Fig. 3:**
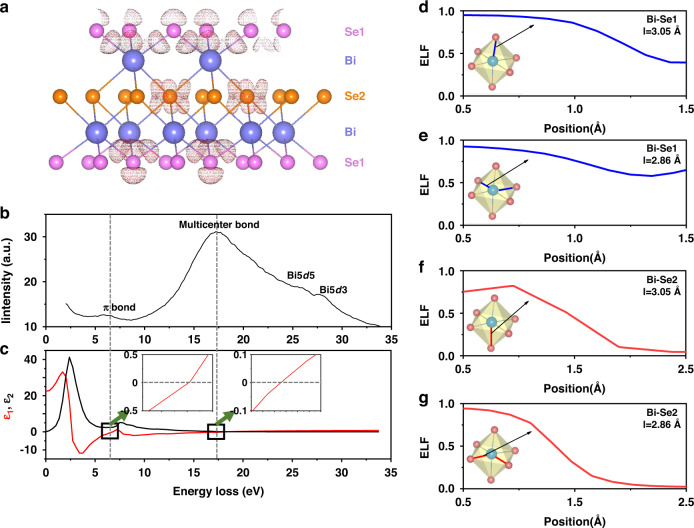
Theoretical calculation and experimental characterization of r-Bi_2_Se_2.4_ bonding. **a** Electron density difference map of r-Bi_8_Se_12_. **b** The EELS spectrum of r-Bi_2_Se_2.4_. **c** The real (ε_1_) and imaginary (ε_2_) parts of r-Bi_8_Se_12_ calculated by DFT. The inset is an enlarged view of the area where ε_1_ is close to zero. This indicates that there is plasma oscillation when ε_1_ passes through the zero point with a positive slope. Electronic localization function of r-Bi_8_Se_12_: **d** the Se1-Bi long bond, **e** Se1-Bi short bond, **f** Bi-Se2 long bond, and **g** Bi-Se2 short bond

In order to further characterize the bonding structure, we established an r-Bi_8_Se_12_ unit cell and performed theoretical calculations. As shown in Fig. 3a, the electron density difference map can be divided into two parts: the surface and the body. The first and third layers of Se atoms have π electrons side by side, indicating the formation of surface π bonds^33^. In a QL layer, each Bi and six Se form six bonds, in which the bond length of four long bonds are 3.05 Å, and the bond length of two short bonds are 2.86 Å, forming an octahedron. The electron-rich regions around the long and short bonds gradually disappear from the first layer of Se atoms (Se1) to the first layer of Bi atoms (Bi), and then to the second layer of Se atoms (Se2). This indicates that a bond of electron delocalization forms. The similar results also appear in the Electron Localization Function (ELF) spectrum. From Se1 to Bi1, and then to Se2, the ELF value of the short bond is changed from 0.64 to 0.02 (Fig. 3e, g), and the ELF value of the long bond is changed from 0.39 to 0.05 (Fig. 3d, f). These results indicate that the valence electrons in the r-Bi_8_Se_12_ unit cell are neither delocalized like the π-bond on the surface nor localized like the conventional covalent bond, but in a semi-delocalized state. This is the typical characteristic of electron-deficiency multicenter bonds (EDMB)^34^. The EDMB refers to the chemical bond formed by the superposition of the atomic orbitals of three or more atoms in a material system with a number of valence electrons less than the coordination number^35^. Low ionization, low hybridization, and low saturation are the three necessary principles for the formation of electron-deficiency multicenter bond. When Bi forms an octahedral configuration with the surrounding 6 Se atoms, the valence electron number (5) of Bi is smaller than the coordination number (6). Accordingly, the valence electrons of Bi are not owned by a single bond, but are simultaneously shared by six surrounding Bi-Se bonds. This leads to the formation of semi-delocalized EDMB instead of localized covalent bonds between Bi and neighboring Se. Indeed, the researchers also discovered the electron-deficiency multicenter bond in heavy-metal chalcogenides such as c-PbTe^36^, r-Bi_2_Te_3_^36^, r-Sb_2_Te_3_^37^, and c-Ge_2_Sb_2_Te_5_^38^ through density functional theory. They believe that this bond has a longer bond length and a higher degree of electron delocalization than conventional covalent bonds. These results are in good agreement with our calculations.

We used low energy electron loss spectroscopy (EELS) to further characterize the bonds in r-Bi_2_Se_2.4_. As shown in the EELS of r-Bi_2_Se_2.4_ (Fig. 3b), there are three characteristic peaks located at 26–28 eV, 17 eV, and 7 eV. The characteristic peaks of electron loss at 26–28 eV are consistent with the Bi5*d*^5^ and Bi5*d*^3^ peaks of the XPS of Bi element (details in SI). Therefore, these peaks can be attributed to Bi5*d*^5^ and Bi5*d*^3^ interband transitions. In the valence band spectrum of the Bi_2_Se_2.4_ film (details in SI), there are no peaks at both around 7 eV and 17 eV. However, in the real part spectrum of the dielectric function (Fig. 3c), the position of *ε*_1_ = 0 corresponding to the plasma energy appeared at around 7 eV and 17 eV. These indicate that the two peaks of around 7 eV and 17 eV were not caused by interband transitions, but by two plasma oscillations. Based on the previous studies, the plasma oscillation of around 7 eV originates from surface π delocalized electrons^39^, and the plasma oscillation of around 17 eV originates from the in-body electrons between delocalized and localized states^40^. Therefore, two peaks of 7 eV and 17 eV can be attributed to the surface π bond and the multicenter bond formed in r-Bi_2_Se_2.4_, respectively, which is consistent with the calculated results above. In addition, we calculated the Bohr radius of the amorphous and crystalline r-Bi_2_Se_2.4_ film through the formula in Supplementary information C3 to characterize the distance between the positive charge and the bound electron. The Bohr radius of amorphous Bi_2_Se_2.4_ is only 127 pm. However, the Bohr radius of r-Bi_2_Se_2.4_ is rapidly increased to 210 pm. This is in good agreement with the theoretical calculations and EELS results. This proves that the high *ε*_*opt*_ of r-Bi_2_Se_2.4_ originates from the formation of electron-deficiency multicenter bond, which increases the Bohr radius and electron delocalization, resulting in a strong electron-polarization effect.

### What are the high-*ε*_*opt*_ materials?

In previous studies on chalcogenide phase change alloys, Wuttig et al. reported the important role of low ionization and low hybridization in the formation of semi-delocalized resonance bonds^41^. Our results of Bi_2_Se_2.4_ show that the low saturation caused by the increase of the coordination number also plays an important role in the generation of electron-deficiency multicenter bonds. Therefore, we proposed the following three microscopic principles for designing high-*ε*_*opt*_ crystals with electron-deficiency multicenter bonds (details in SI). (1) Low degree of ionization (*D*_*i*_). *D*_*i*_ is the difference between the sum of the *s*-, *p*-, *d-*orbital radii of cations *M* and anions *N*^41^ (Eq. 3). The greater the *D*_*i*_, the more likely *M* and *N* atoms are to form ionic bonds by gaining or losing electrons. (2) Low degree of hybridization (*D*_*h*_). The *D*_*h*_ is defined as the reciprocal of the sum of the difference between the outermost *d-*orbital and innermost *s-*orbital radii of cations *M* and anions *N*^41^ (Eq. 4). The greater the *D*_*h*_, the more likely the *M* and *N* atoms to form a covalent bond by sharing valence electrons. (3) Low degree of saturation (*D*_*s*_). The *D*_*s*_ is the ratio of the coordination number of atoms under the 8-N rule to the actual coordination number^37^ (Eq. 3). *No*. refers to the number of valence electrons of the atom. The actual coordination number is the number of nearest neighbors of the reference atom in the actual crystal structure.

3 $$D_i = \left[ {\left( {r_s^M + r_p^M + r_d^M} \right) + \left( {r_s^N + r_p^N + r_d^N} \right)} \right]/\frac{{r_M + r_N}}{2}$$

4 $$D_{{{\mathrm{h}}}} = \left[ {\left( {r_d^M - r_s^M} \right) + \left( {r_d^N - r_s^N} \right)} \right]^{ - 1}/\frac{{r_M + r_N}}{2}$$

5 $$D_s = \frac{{CoN_{(8 - N{{{\mathrm{o}}}}.)rule}}}{{CoN_{actual}}}$$

**Fig. 4:**
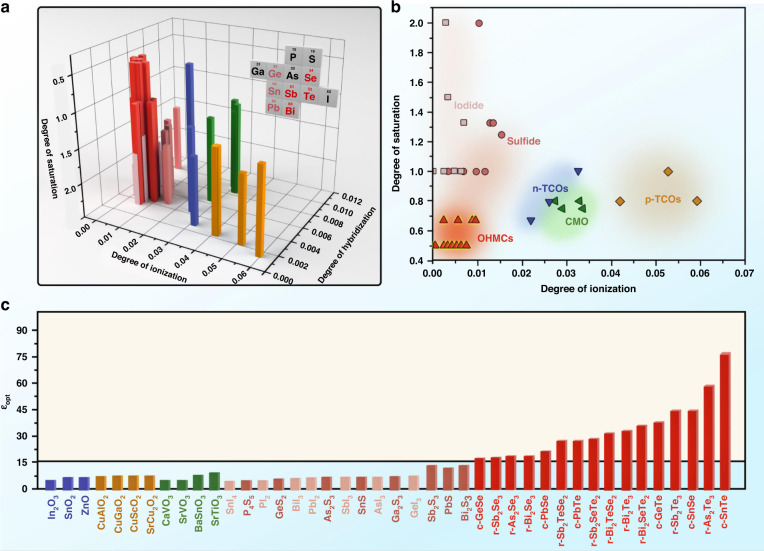
Map of high-*ε*_*opt*_ material. **a** 3D graphs of the ionization, hybridization, and saturation of different materials. The inset in the upper right corner shows the elements of the candidate compounds. Five types of system are shown: heavy-metal chalcogen compounds of type *IV*_1_*VI*_1_ and *V*_2_*VI*_3_ (red), n-type transparent conductive oxide (blue), p-type transparent conductive oxide (orange), correlated metal oxonates (green), sulfide (pale-red) and iodide (pink). **b** 2D graphs of the ionization and saturation of the same material. The material in the bright-red area meets all the three principles. The material in the pale-red area has a high degree of hybridization. Materials outside the bright-yellow and pale-yellow area are highly ionized or highly saturated. **c** *ε*_*opt*_ of the same material. The bright yellow materials that meet all the three principles are high-*ε*_*opt*_ with *ε*_*opt*_ > 15

To predict more high-*ε*_*opt*_ candidate materials, we used the above three microscopic principles to establish a three-dimensional coordinate system (Fig. 4a), and predicted the possible compounds in the figure. We selected the cations *M* and anions *N* in the compound based on the following criteria. (1) To obtain a low degree of ionization, degree of hybridization, and a high coordination number equal to or greater than 6, the electronegativity of anions *N* should be small, the number of periods should be large, and the number of valence electrons should be large. Therefore, the heavy atoms S, Se, and Te of the *VIA* group were mainly selected as anions *N* (Fig. 4a). The heavier I atom was also selected for the comparison. (2) To obtain low degree of ionization, degree of hybridization, and unsaturated bond, the electronegativity and period number of cations *M* should be as large as possible, and the number of valence electrons should be less than 6. Therefore, the heavy atoms Sn, Pb, Sb, and Bi of the *IVA* and *VA* groups were mainly selected as the cations *M* (Fig. 4a). (3) To predict more high-*ε*_*opt*_ candidate materials, the atomic composition, stoichiometric ratio, and crystal structure of the materials in this paper were diverse. These materials could be prepared and were all reported in existing literatures.

In Fig. 4a, the degree of ionization, degree of hybridization, and degree of saturation of r-Bi_2_Se_3_ are only 7.5 E + 03, 4.7 E − 03, and 0.5. Therefore, the electrons will not locally form an ionic bond around an atom, nor will it locally form a covalent bond between two atoms. They are shared by multiple atoms, forming highly delocalized electron-deficiency multicenter bonds. Since the increase in the degree of ionization, degree of hybridization, or degree of saturation will increase the localization of valence electrons near the ionic substance, typical multicenter bond materials should be mainly formed in a small area near r-Bi_2_Se_3_. A side view of Fig. 4a was provided in Fig. 4b to show it more clearly. The bright-red area is the low degree of ionization, low degree of hybridization and low degree of saturation area. That is, it meets all the three principles. The material in the pale-red area has a high degree of hybridization. Materials outside the red area are highly ionized or highly saturated. Figure 4c shows the *ε*_*opt*_ of all the materials in Fig. 4b (details in SI). The results are in agreement with our prediction. The materials that meet all the three principles are high-*ε*_*opt*_ materials, and materials that do not meet any of the principles are low-*ε*_*opt*_ materials. Among these low-*ε*_*opt*_ materials, some materials (In_2_O_3_:Sn) have a high degree of ionization, and some materials (SrVO_3_) have a high degree of hybridization. Iodine and chalcogen compounds, such as BiI_3_ and Bi_2_S_3_, have the low degree of ionization and degree of hybridization. However, since their degrees of saturation are too great, they do not have a high *ε*_*opt*_. These results prove that low degree of ionization, low degree of hybridization and low degree of saturation are the three necessary principles for the formation of high-*ε*_*opt*_ crystals.

In Fig. 4, high-*ε*_*opt*_ materials are mainly heavy-metal chalcogenides in octahedral configuration including r-Bi_2_Se_3_, r-Sb_2_Te_2_Se, r-As_2_Te_3_, r-Bi_2_STe_2_, r-Bi_2_Te_3_, r-Sb_2_Te_3_, c-SnTe, c-PbSe, c-PbTe. Their phase characteristics and chemical formula can be described as: (1) r-M_2_N_3_ with rhombohedral structure (M = Bi, Sb, As, etc., N = Se, Te, Se+Te); (2) c-M_1_N_1_ with cubic structure (M = Pb, Sn, etc., N = Se, Te, Se+Te).

### Can high *ε*_*opt*_ really break the far-infrared transparent and conductive trade-off?

**Fig. 5:**
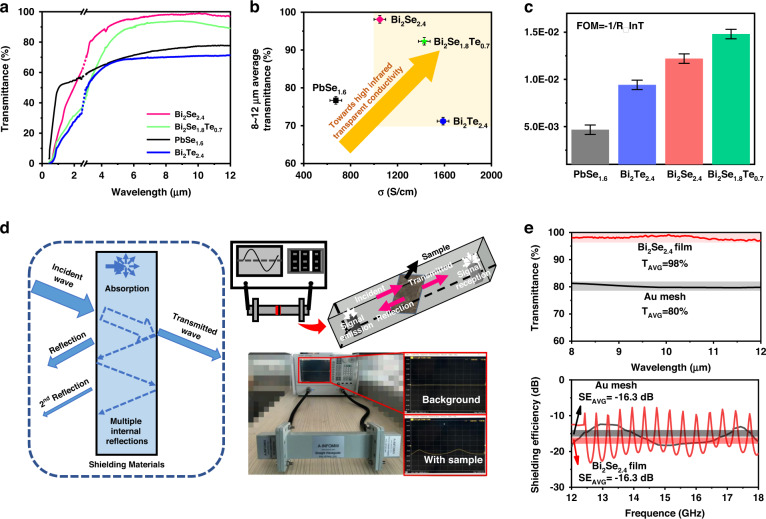
Far-infrared transparent conductive properties and electromagnetic shielding efficiency of high-*ε*_*opt*_ materials. **a** Far-infrared transmission spectra, **b** conductivity, average transmittance, and **c** FOM for r-Bi_2_Se_2.4_, r-Bi_2_Te_2.4_, r-Bi_2_Se_1.8_Te_0.7_, r-PbSe_1.6_. We have removed the 2.5–3 μm data due to the presence of instrument-generated miscellaneous peaks and connected the data with dashed lines for aesthetic reasons. **d** Diagram of electromagnetic shielding principle (left) and self-built electromagnetic shielding efficiency test system (right). **e** Far-infrared transmittance and electromagnetic shielding efficiency for Bi_2_Se_2.4_ film and Au mesh

To verify that the high-*ε*_*opt*_ material is a potential FIRTC, we tested the infrared transmittance and conductivity at room temperature of r-Bi_2_Se_2.4_ with Se vacancies and different film thicknesses (details in SI). The results are shown in Fig. 5a, Fig. S7, and Fig. 5b. The conductivity of r-Bi_2_Se_2.4_ film is 1049.4 S/cm. The transmittances of 3–5 μm and 8–12 μm in the two atmospheric windows are as high as 85% and 98%, respectively. These indicators have exceeded the application requirements of infrared transparent conductive film (conductivity is 1000 S/cm, transmittance is 70%). This shows that r-Bi_2_Se_2.4_ film is a wide-band infrared TC. In Figure S7, the *λ*_*p*_ of traditional transparent conductive materials, such as n-type oxides (ITO), p-type oxides (CuScO_2_), ultra-thin metals (Ag), and correlated metal oxonates (SrVO_3_), are all less than 5 μm. The far-infrared transparent conductive properties of r-Bi_2_Se_2.4_ film are not possessed by other traditional materials. Therefore, it can be regarded as the first FIRTC.

We first explain the reasons for the high conductivity of r-Bi_2_Se_2.4_ film. We find that the Hall coefficient of the r-Bi_2_Se_2.4_ film is negative (R_H_ = −0.05) through the Hall effect test (details in SI). This indicates that the conduction mode of the film is electronic conduction. The electrons in the film mainly come from Se vacancies. Se vacancies, as a kind of shallow energy level defect, can generate free electrons through thermal excitation at room temperature^42^. Studies revealed that Se vacancies in bismuth selenide films are mainly derived from the evaporation of Se atoms during film sputtering^43^. Because among all types of point defects (vacancies, interstitial atoms, and anti-site atoms) Se vacancies have the lowest formation energy and are most easily formed in selenium-deficient bismuth selenide^44^. In Table S1, the electron concentration of Bi_2_Se_2.4_ film is much smaller than that of ITO, and its relaxation time is much larger than that of ITO. Also, both materials have similar effective masses^43,45^. Therefore, Bi_2_Se_2.4_ has a high conductivity because of its high relaxation time. The high relaxation time of Bi_2_Se_2.4_ mainly comes from two aspects. One is that r-Bi_2_Se_3_ crystal has higher intrinsic mobility, which can be attributed to its smaller effective mass and larger deformation potential energy^46^. The second is that the electron concentration of r-Bi_2_Se_2.4_ film is lower. This reduces the collision between electrons. The detailed discussion is provided in Supplementary information B8.

Next, we discuss the reasons for the excellent far-infrared transmission of r-Bi_2_Se_2.4_ film. The frequency corresponding to *ε*_*r*_ = 0 in the dielectric real part spectrum is taken as the plasma frequency *ω*_*p*_ to obtain *λ*_*p*_ (details in SI). In Fig. S7, the *λ*_*p*_ of r-Bi_2_Se_2.4_ film (21.4 μm) is much larger than that of ITO (1.5 μm), which is mainly due to the great shielding effect from high *ε*_*opt*_ of r-Bi_2_Se_2.4_. This weakens the coulomb effect of the ion on the free electrons, which in turn increases the *λ*_*p*_ (Eq. 1). λ_p_ of r-Bi_2_Se_2.4_ is 21.4 μm, which is far greater than 12 μm. Hence, its interband transition absorption hardly occurs in the far-infrared band (8–12 μm) we focus on in this article. In addition, it is shown that the band gap (*E*_*g*_) and transverse optical branch vibration frequency (*ω*_*TO*_) of the r-Bi_2_Se_2.4_ film are 0.55 eV (details in SI) and 0.03 eV^47^, respectively. This makes the interband transition absorption and lattice vibration absorption of the r-Bi_2_Se_2.4_ film in the band less than 2.5 μm and in the band centered at 41 μm, respectively, far away from the far-infrared band. These are the reasons for excellent far-infrared transmission of the r-Bi_2_Se_2.4_ film.

To further optimize the phase structure and atomic composition for better far-infrared transparent conductive properties, we prepared other typical candidate materials under similar experimental condition as r-Bi_2_Se_2.4_, including r-Bi_2_Te_2.4_, r-Bi_2_Se_1.8_Te_0.7_, and c-PbSe_1.6_. Their composition, structure, and transparent conductive properties under different thicknesses are shown in Supplementary information. Their transmittance and electrical conductivity are shown in Fig. 5a, b. From Fig. 5a, the transmittance of all four film samples in 8–12 μm is all over 60%, with that of r-Bi_2_Se_2.4_ and r-Bi_2_Se_1.8_Te_0.7_ exceeding 90%, which is attributable to the large *λ*_*p*_, and the lack of lattice vibration absorption and interband transition absorption. In 3–8 μm, these four samples still have high transmittance. In 0.4–2.5 μm, however, the transmittance decreases to 0 below the interband transition absorption wavelength. All materials have far-infrared transparent and conductive properties, indicating that increasing the *ε*_*opt*_ is an effective and universal strategy to break the trade-off between far-infrared transparency and conductivity. The Hall coefficients of all these films are negative through the Hall test. This indicates that their main carriers are free electrons. We used a figure of merit (FOM) to quantitatively describe the transparent conduction properties of the material (details in SI), and the results are shown in Fig. 3c. Both average transmittance and conductivity of c-PbSe_1.6_ are lower than that of other rhombohedral films. This indicates that the rhombohedral phase is more favorite to high transparent conductive properties. This is because the rhombohedral phase is mainly composed of group VA elements and VIA elements, and the cubic phase is mainly composed of group IVA elements and VIA elements. The bond saturation of the former (0.50) is lower than that of the latter (0.67), which favors the high *ε*_*opt*_. In all rhombohedral films, the FOM of r-Bi_2_Se_1.8_Te_0.7_ is significantly greater than that of both r-Bi_2_Se_2.4_ and r-Bi_2_Te_2.4_. This is because r-Bi_2_Se_1.8_Te_0.7_ has not only a high *ε*_*opt*_ (27.0), but also a moderate carrier concentration (1.2 E + 20 cm^−3^). Therefore, among all the high-*ε*_*opt*_ chalcogenides, the heavy-metal selenium telluride in octahedral configuration has the best comprehensive properties.

An important application of FIRTC is the electromagnetic shielders for far-infrared windows. Since there was no far-infrared transparent conductive material in the past, the traditional electromagnetic shielder for the far-infrared window is mainly a metal mesh such as Au. In this paper, to explore the application potential of the r-Bi_2_Se_2.4_ film, we used our own assembled test device (Fig. 5d) to estimate the electromagnetic shielding efficiency. In Fig. 5d, the left diagram describes the interaction of electromagnetic waves and shielding materials. When electromagnetic waves are incident on the surface of the shielding material, a portion of the electromagnetic waves are reflected on the outer surface. Then, the remaining electromagnetic waves enter the material and are reflected multiple times between the two interfaces. In this process, the energy of light is continuously attenuated. Eventually, the unabsorbed electromagnetic waves pass through the shielding material. The right diagram describes the working principle and actual image of the electromagnetic shielding test system. The results are shown in Fig. 5e. The performance of the traditional Au mesh is provided for the comparison. As shown in Fig. 5e, the electromagnetic shielding efficiency of the r-Bi_2_Se_2.4_ film is SE = −16.3 dB, which is very close to that of the Au mesh (SE = −15.7 dB). The average transmittance of the r-Bi_2_Se_2.4_ film in the 3–5 and 8–12 μm bands is 86 and 98%, respectively, which are greater than that of the Au mesh (T = 87%, 80%)^48^. Therefore, the new far-infrared transparent conductive film in this paper has good application prospects, and the bottleneck problems of poor imaging quality and complex processing of traditional far-infrared electromagnetic shielding mesh is expected to be solved.

Based on good electrical conductivity and infrared transmission, our FIRTC may also be applied to photodetectors and invisible sensors. As an important part of the optical fiber communication system, the photodetector is a component that converts optical signals into electrical signals. It is widely applied in optical communication^12^, laser guidance^19^, biosensing^20^, etc. If the transparent electrode is replaced from the traditional ITO to our FIRTC, the applicable band of the photodetector can be complemented to far-infrared region, which will promote the development of far-infrared search and guidance technology. The invisible sensor^15^ is a new type of sensor that can effectively hide its own thermal and electronic signals and it can be applied to infrared thermal camouflage, infrared photoelectric countermeasures, and other fields. If our FIRTC is applied to the camouflage shell, the invisible sensor is expected to disguise the target under multiple physical fields such as infrared light, electricity, and heat, which will facilitate the development of technologies such as infrared camouflage and infrared remote sensing.

## Discussion

In conclusion, we prove that increasing the optical dielectric constant is an effective and universal strategy to break the trade-off between infrared transparency and conductivity through experiments and theoretical calculations. We have obtained the first family of far-infrared TCs. Using this type of materials, we have developed the first “continuous film” type far-infrared electromagnetic shielder. Unlike the well-known transparent conductive material ITO (indium-tin oxide), which is used in the visible band, the material we have developed is a transparent conductive material used in the far-infrared band, called “FIRTC”. We expect that this “FIRTC” can be applied to the technical fields that are beyond the reach of traditional visible ITO, such as photodetectors, invisible sensors, etc. This will lead to more new research into optoelectronic physics, materials, and devices.

## Materials and methods

### Material preparation

Using magnetron sputtering, we simultaneously deposited thin films on single-crystal Si, polycrystalline zinc sulfide and glass at the same time. Among them, single-crystal Si is used for structural testing of thin film samples, polycrystalline zinc sulfide for infrared transmission spectrum and electrical tests, and glass for visible light transmission spectrum tests. Before thin film deposition, the substrate was sequentially placed in acetone, alcohol, and deionized water for ultrasonic vibration cleaning, and then they were introduced into the sputtering vacuum chamber (base pressure 4*10^−4^ Pa). To obtain Bi_2_Se_x_, Bi_2_Te_x_, Bi_2_Se_x_Te_y_, PbSe_*x*_, and In_2_O_3_:Sn films, the high-purity Bi_2_Se_3_ (99.99%), Bi_2_Te_3_ (99.99%), PbSe (99.99%), and In_1.7_Sn_0.3_O_3_ (99.99%) targets are sputtered in a pure Ar atmosphere. The Bi_2_Se_x_Te_y_ was obtained by co-sputtering Bi_2_Se_3_ and Bi_2_Te_3_ targets. During the sputtering process, the deposition parameters were set as follows: substrate temperature: 200–300 °C; substrate bias: floating; RF power applied to the targets: 60–90 W; pressure: 1 Pa; deposition time: 15–300 s. In addition, a tube furnace was used to anneal the obtained film at a high temperature in an Ar atmosphere. To prevent the Se in the film from evaporating, we placed a small amount of Se particles in the tube furnace to create a Se atmosphere. The heating rate was 5 °C/min, the annealing temperature was 200–400 °C, and the holding time was 10–30 min. The detailed preparation and annealing conditions, and the thickness of each film are listed in Supplementary information G1.

### Material characterization

Surface profiler (Bruker, Inc., DEKTAK PROFILER 150) was used to characterize the thickness of the films. X-ray photoelectron spectroscopy (VG Scientific Ltd, Inc., VG ESCALAB MK II) and energy dispersive spectrometer equipped in a field-emission scanning electron microscope (SU8010, Hitachi, Tokyo, Japan) were used to measure the composition of the films. Atomic force microscope (Bruker, Inc., Dimension Icon) was used to characterize the surface morphology of the films. The high-resolution transmission electron microscopy (JEM-2100F, JEOL, Tokyo, Japan), grazing-incidence X-ray diffraction (Bruker, Inc., D8-tools XRD), Raman (B&W TEK, Inc., GlacierTM T), Electron Energy-loss spectroscopy (EELS) (JEOL, Inc., JEM-ARM300F) and XPS valence band spectra were used to characterize the structure and chemical bonding of the films. Ultraviolet-visible-near infrared photometer (Perkin Elmer, Inc., Lambda 950) and Fourier transform infrared spectroscopy (Perkin Elmer, Inc., Spectrum One) were used to test the transmittance of the film at 200–2500 nm and 2500–16000 nm. The electrical properties of the film such as conductivity, sheet resistance, carrier concentration, and mobility were measured by Hall tester (CH Electronic Devices, Inc., CH-50), detailed in supporting information.

### First-principles calculations

Our first-principles calculations for the electronic structure of Bi_2_Se_3_ with rhombohedral phase (space group R-3m) were performed using the VASP module, on the basis of the density functional theory (DFT) combined with the projector augmented wave (PAW) method. The exchange correlation effect was described by generalized gradient approximation (GGA) in Perdew–Burke–Ernzerhof (PBE) functional form. The structure was fully relaxed until the total energy converges to below than 10^−4^ eV, in which a kinetic energy cut-off for the plane wave of 550 eV was used to minimize the local energy. The optimized Bi_2_Se_3_ geometrical structure with a lattice parameter of 10.42 Å, which is consistent with the experimental value (10.40 Å)^33,49^.

### Spectrum fitting

To obtain the dielectric function spectrum of the film, we used the Drude-Lorentz model to fit the infrared transmission spectrum. The formulas are

6 $$\varepsilon \left( E \right) = \varepsilon _\infty - \frac{{E_p^2}}{{E^2 - i{{{\mathrm{{\Gamma}}}}}_DE}} + \mathop {\sum }\limits_{j = 1}^n \frac{{S_jE_{0j}^2}}{{E_{0j}^2 - E^2 + iE{{{\mathrm{{\Gamma}}}}}_i}},\;n = 1,2,3$$

7 $$n\left( E \right) = \frac{1}{{\sqrt 2 }}\left[\sqrt {\varepsilon _1^2\left( E \right) + \varepsilon _2^2\left( E \right)} + \varepsilon _1\left( E \right)\right]^{1/2}$$

8 $$k\left( E \right) = \frac{1}{{\sqrt 2 }}\left[\sqrt {\varepsilon _1^2\left( E \right) + \varepsilon _2^2\left( E \right)} - \varepsilon _1\left( E \right)\right]^{1/2}$$

where *ε*_1_ and *ε*_2_ are the real and imaginary parts of the dielectric function. *S*, *E*_0_, and *Г* are the amplitude, the energy of the transition center, and the broadening term, respectively. *ε*_*∞*_ is the high-frequency dielectric constant, and *E* is the incident photon energy. *E*_*p*_ is the plasma energy, and proportional to the free electron concentration of the material. *Г*_D_ is the relaxation energy. The transmittance of the substrate film can be obtained from Eqs. (9) and (10):

9 $$\left[ {\begin{array}{*{20}{c}} B \\ C \end{array}} \right] = \left[ {\begin{array}{*{20}{c}} {\cos \delta _1} & {\frac{{{{\mathrm{i}}}}}{{\eta _1}}\sin \delta _1} \\ {{{{\mathrm{i}}}}\eta _1\sin \delta _1} & {\cos \delta _1} \end{array}} \right]\left[ {\begin{array}{*{20}{c}} 1 \\ {\eta _2} \end{array}} \right]$$

where the phase difference is $\delta _1 = \frac{{2\pi }}{\lambda }\left( {n + ik} \right)d$, the film thickness is *d*, the admittance of the film $\eta _1 = N = n - ik$, and the admittance of the substrate is *η*_*2*_.

10 $$T = \frac{{4n_0\eta _2}}{{\left( {B + C} \right)\left( {B + C} \right)}}$$

Substituting the expressions of ε_1_(*E*) and ε_2_(*E*) into Eqs. (7) and (8), and substituting the obtained expressions into Eqs. (9) and (10), the transmittance function used to fit the experimental data is as follows:

11 $$T\left( E \right) = \frac{{[n\left( E \right) - 1]^2 + k^2\left( E \right)}}{{[n\left( E \right) + 1]^2 + k^2\left( E \right)}}$$

The optical dielectric constant *ε*_*opt*_ of the film and the absorption edge (*λ*_*p*_) are obtained through the dielectric function spectrum, detailed in the supplementary information.

### Electromagnetic shielding test platform and performance evaluation

An electromagnetic shielding test system was established with a vector network analyzer (Agilent, Inc., N523xA PNA-L) as the core. We used this system to obtain the electromagnetic shielding effectiveness of the film sample in the 12–18 GHZ frequency band, more details of the device and test are shown in the supplementary information.

## Supplementary information

Supplementary Information
