# Supplementary material for: Far-infrared transparent conductors
Source: Light Sci Appl. 2023 Apr 21;12:98. doi: 10.1038/s41377-023-01139-w (PMC10121591; doi:10.1038/s41377-023-01139-w)
Supplement: Supplementary file 1 — Supplementary Information [file 41377_2023_1139_MOESM1_ESM.docx]

**SUPPLEMENTARY INFORMATION**

**Far-infrared transparent conductors**

Chaoquan Hu^1^,*, Zijian Zhou^1^, Xiaoyu Zhang^1^, Kaiyu Guo^1^, Can Cui^1^, Yuankai Li^1^, Zhiqing Gu^2^, Wei Zhang^1,3^, Liang Shen^4^,* and Jiaqi Zhu^5^,*

*^1^ State Key Laboratory of Superhard Materials, Key Laboratory of Automobile Materials of Ministry of Education, School of Materials Science and Engineering, Jilin Provincial International Cooperation Key Laboratory of High-Efficiency Clean Energy Materials, Jilin University, Changchun, 130012, PR China*

*^2^ College of Information Science and Engineering, Jiaxing University, Jiaxing 314001, PR China*

*^3^ Electron Microscopy Center, Jilin University, Changchun, 130012, PR China*

*^4^ State Key Laboratory of Integrated Optoelectronics, College of Electronic Science and Engineering, International Center of Future Science, Jilin University, Changchun 130012, PR China*

*^5^ National Key Laboratory of Science and Technology on Advanced Composites in Special Environments, Harbin Institute of Technology, Harbin, 150080 P. R. China*

**This file includes:**

Figures S1-S14

Tables S1-S8

References 1-43

***Corresponding authors:** [cqhu@jlu.edu.cn](mailto:cqhu@jlu.edu.cn) (C. Hu); [shenliang@jlu.edu.cn](mailto:shenliang@jlu.edu.cn) (L. Shen); [zhujq@hit.edu.cn](mailto:zhujq@hit.edu.cn) (J. Zhu)

**A Structure and composition**

*A1 X-ray diffraction*

Grazing Incidence X-ray Diffraction (GIXRD) were performed for all films. The GIXRD of five typical samples are shown in Fig. S1. The significant diffraction peaks indicates that Bi_2_Se_2.4_, Bi_2_Te_2.4_, Bi_2_Se_1.8_Te_0.7_, PbSe_1.6_ and In_1.7_O_3_:Sn_0.3_ films have the crystalline structure of rhombohedral phase^1^, rhombohedral phase^2^, rhombohedral phase^3^, cubic phase^4^ and cubic phase^5^, respectively.

*A2 Atomic force microscope*

Atomic Force Microscope (AFM) were performed for all films. The three-dimensional AFM of typical samples (Bi_2_Se_2.4_, Bi_2_Te_2.4_, Bi_2_Se_1.8_Te_0.7_ and PbSe_1.6_) are shown in Fig S2. These films are smooth. Root mean square roughness (RMS) of Bi_2_Se_2.4_ (d = 42 nm ± 3 nm), Bi_2_Te_2.4_ (d = 41 nm ± 3 nm), Bi_2_Se_1.8_Te_0.7_ (d = 42 nm ± 3 nm) and PbSe_1.6_ (d = 40 nm ± 3 nm) were determined to 3.35 nm, 1.34 nm, 1.6 nm and 0.83 nm, respectively.

*A3 DOS and valence band spectra of r-Bi_2_Se_2.4_*

To assign the unknown peaks of EELS, the valence band spectrum of r-Bi_2_Se_2.4_ is shown in Fig. S3. The Bi5*d*5 and Bi5*d*3 peaks of the XPS of Bi element are located at 26.2 eV and 27.0 eV, respectively. These are in good agreement with the electron loss characteristic peaks around 26-28 eV in EELS. Therefore, this peak can be attributed to Bi5*d*5 and Bi5*d*3 interband transitions^6^. In the valence band spectrum of the Bi_2_Se_2.4_ film (Fig. S3), there are no peaks at both around 7 eV and 17 eV. This indicates that the two peaks around 7eV and 17 eV in EELS are not caused by the interband transitions.

*A4 Chemical composition*

The chemical composition of the film was tested by a photoelectron spectrometer model VG ESCA LAB MKⅡ. Before the XPS test, Ar^+^ was used to etch the surface of the film for 120 s to remove the adsorbed oxygen and carbon on the surface. The high-resolution scanning maps of the elements were provided to analyze the chemical composition content more accurately. The chemical composition of the film is calculated by the following formula^7^. We took the Bi_x_Se_y_ film as an example and used the integrated area of the Bi 4*f* peak and Se 3*d* peak to calculate the relative content of Bi and Se. The formulae are

$C_{B\text{i}}=\frac{A_{B\text{i}}/S_{B\text{i}}}{A_{B\text{i}}/S_{B\text{i}}+A_{S\text{e}}/S_{S\text{e}}}$ (1)

$C_{Se}=\frac{A_{S\text{e}}/S_{S\text{e}}}{A_{B\text{i}}/S_{B\text{i}}+A_{S\text{e}}/S_{S\text{e}}}$ (2)

Where *C*_Bi_ and *C*_Se_ represent the relative content of Bi and Se, respectively. *A*_Bi_ and *A*_Se_ represent the integrated areas of the Bi 4*f* peak and Se 3*d* peak, respectively. *S*_Bi_ and *S*_Se_ represent elemental sensitivity factors of Bi and Se, respectively. Since the X-ray source is Al *K*_α_ (1486.6 eV), *S*_Bi_ and *S*_Se_ are 9.14 and 0.85, respectively. The XPS high-resolution elemental scanning maps of Bi_x_Te_y_, Bi_x_Se_y_Te_z_, Pb_x_Se_y_ and In_x_Sn_z_O_y_ films are shown in Fig. S6. Their chemical composition is determined by the same method. The used element sensitivity factors are as follows: *S*_Te_=5.705, *S*_Pb_=8.329, *S*_In_=4.359, *S*_Sn_=4.725, *S*_O_=0.711. The results are shown in Table S1.

The effect of annealing temperature and protection atmosphere (selenium gas) on the chemical composition of bismuth selenide films are shown in Fig. S4 and Fig. S5, respectively. During the annealing process without selenium gas (pure argon atmosphere), as the annealing temperature increased from 200 °C to 400 °C, the Se content in the film decreased from 50.7% to 47.0%, which was attributed to the evaporation of Se in the film caused by an increase in temperature. However, in the presence of selenium gas, as the annealing temperature increases from 200 °C to 400 °C, the Se content in the film increases from 54.9% to 64.7%, which is attributed to the fact that the temperature increase promotes the incorporation of selenium into the film, which induces Se_Bi_ antisite and/or interstitial defects. Therefore, we can control the chemical compositions and improve the crystallinity of the films by annealing temperature and selenium atmosphere. The chemical compositions of other thin films of bismuth telluride, lead selenide, and bismuth selenide telluride can be controlled by similar methods.

In this paper, the compositions of the four materials with the best performances are Bi_2_Se_2.4_, Bi_2_Te_2.4_, Bi_2_Se_1.8_Te_0.7_ and PbSe_1.6_. They were all obtained at an annealing temperature of 300°C under a protective atmosphere of selenium or/and tellurium.

*A5 Electron energy loss spectroscopy test*

We obtained electron energy loss spectroscopy (EELS) of crystalline r-Bi_2_Se_2.4_ thin films with a spherical aberration-corrected transmission electron microscope model JEM-ARM300F and a model TESCAN multifunctional accessory. We analyzed our obtained EELS results by means of an energy loss function^8^. The differential inelastic scattering cross section of a material can be characterized by the complex dielectric function *ε(q,ω)*, which is expressed as follows:

$\frac{d^{2}\sigma}{d\omega d\Omega}\approx\frac{1}{q^{2}}\mathrm{Im}[\frac{-1}{\varepsilon(q,\omega)}]$ (3)

where $\varepsilon(q,\omega)=\varepsilon_{1}(q,\omega)+i\varepsilon_{2}(q,\omega)$ and $\mathrm{Im}[\frac{-1}{\varepsilon(q,\omega)}]=\frac{\varepsilon_{2}}{\varepsilon_{1}^{2}+\varepsilon_{2}^{2}}$ are the energy loss function. When the energy loss function passes through the energy range of plasma oscillation, *ε_2_* has a weak peak, and *ε_1_* passes through the zero point with a positive slope.

**B Electrical and optical characterization**

*B1 Electrical properties*

Hall electrical characteristics test were performed for all films. All film samples were tested five times, where the two greatest and smallest values were removed and the remaining three data were used to calculate the mean value and error. The Hall coefficients of Bi_2_Se_2.4_, Bi_2_Te_2.4_, Bi_2_Se_1.8_Te_0.7_, PbSe_1.6_ and In_1.7_O_3_:Sn_0.3_ were -0.054, -0.002, -0.006, -0.458 and -0.004, respectively. The Hall coefficients were all negative, indicating that their carriers were all electrons. The electron concentrations were 1.15 E+20 cm^-3^, 3.37 E+20 cm^-3^, 1.22 E+20 cm^-3^, 1.37 E+20 cm^-3^ and 1.64 E+21 cm^-3^, respectively. The relaxation times were 7.8 E-11 s, 4.0 E-11 s, 9.6 E-11 s, 4.2 E-11 s and 1.1 E-11 s, respectively. The electrical conductivities were 1049 S/cm, 1589 S/cm, 1460 S/cm, 674 S/cm and 1763 S/cm respectively. The conductivities of bismuth selenide thin films and ITO thin films are in good agreement with those of thin films prepared by similar methods in the literature^5^. This indicates that our test results are reliable. Detailed data are shown in Table S2.

*B2 Transmission spectrum*

All thin film samples were tested for transmission from 200-2500 nm and from 2500-16000 nm. During each test, the number of scans was 100 to ensure the reliability of the data, and each sample was tested three times to calculate the mean value and variance of transmittance. To compare with the transmittance data in the literature, we used the general method in the literature^9^ to remove the influence of the substrate on the transmittance, and obtained the transmittance of the free-standing film (without the substrate). The calculation process is $T_{film}=\frac{T_{Coated}}{T_{Uncoated}}$, the coated transmittance is divided by the transmittance of the uncoated substrate. The transmission spectra of Bi_2_Se_2.4_, Bi_2_Te_2.4_, Bi_2_Se_1.8_Te_0.7_, PbSe_1.6_ and In_1.7_O_3_:Sn_0.3_ free-standing films are shown in Fig. S7. In addition, the transmission spectra of low-*λ*_p_ conventional transparent conductive materials are provided. The far-infrared transmission properties of high-*λ*_p_ materials (Bi_2_Se_2.4_, Bi_2_Te_2.4_, Bi_2_Se_1.8_Te_0.7_, PbSe_1.6_) are greater than that of low-*λ*_p_ conventional materials (In_1.7_O_3_:Sn_0.3_).

*B3 Figure of merit for transparent conductivity*

To compare the transparent conductive properties of our films, we adopted the definition of the figure of merit for transparent conductivity (FOM) to evaluate the comprehensive properties of the films^10^. The formula is

(4)

where *T* is the transmission spectrum of the film on ZnS substrate (0<T<1), and R_□_ is the square resistance of the film. The FOM of Bi_2_Se_2.4_, Bi_2_Te_2.4_, Bi_2_Se_1.8_Te_0.7_, PbSe_1.6_ and In_1.7_O_3_:Sn_0.3_ films is listed in Table S2.

*B4 Dielectric function spectrum*

To obtain the dielectric function spectrum of the material, we used the Drude-Lorentz model and fitted the transmission spectrum (detailed in the Methods section). The fitted transmission spectra are shown in Fig S8. The fitted transmission spectrum is in good agreement with the experimental transmission spectrum. The dielectric real part of Bi_2_Se_2.4_, Bi_2_Se_1.8_Te_0.7_, PbSe_1.6_ and Bi_2_Te_2.4_ films in the mid-far infrared band shows normal dispersion, that is, it gradually decreases with the increase of wavelength. This is in good agreement with the results of the literature. Bi_2_Se_1.8_Te_0.7_ and Bi_2_Te_2.4_ show anomalous dispersion in the near-infrared band. This is due to their small band gaps of 0.46 eV (3600 nm) and 0.55 eV (2254 nm). They are in the near-infrared band with strong absorption. The comparison between fitting parameters and experimental parameters are listed in Table S3. The fitted plasma energy and optical gap are in good agreement with our experimental parameters in terms of magnitude and trend.

*B5 ε_opt_*

The polarization process of the dielectric mainly includes electron displacement polarization, atomic polarization, ionic polarization and dipolar polarization. In different frequency ranges, the polarization mechanism of the dielectric is different. For example, under the low-frequency electric field, all of the polarizations above have enough time to respond to the change in the external electric field, and therefore all of these contribute to the static dielectric constant. However, under the high-frequency electric field, only electrons with small mass and low inertia can keep up with the change of high-frequency electric field, while other polarizations have no time to respond. Therefore, only electronic displacement polarization contributes to the dielectric constant. Electron displacement polarization refers to the polarization generated by the displacement of electrons relative to the nucleus in response to the external electric field. The optical dielectric constant *ε_opt_* in our article refers to the dielectric constant in the ultraviolet-visible-near infrared frequency range, which mainly arises from the electronic displacement polarization^11^. The *ε_opt_* of the four materials that we prepared are determined by the dielectric function spectra obtained from transmission spectroscopy simulations. The real part of the dielectric function within 2-2.5 μm is approximately constant. Therefore, we take the average value of the dielectric constant in this band as *ε_opt_*. This method of determining *ε_opt_* has been reported elsewhere^12^.

*B6 Reflection edge*

There are three reported methods of reflection edge (*λ_p_*) measurement. (1) Using the formula $\lambda_{p}=\frac{2\pi C_{0}}{\omega_{p}^{*}}=\frac{2\pi C_{0}}{\sqrt{m^{*}\varepsilon_{0}\varepsilon_{opt}/ne^{2}}}$; (2) Using the frequency corresponding to the maximum slope of the reflection spectrum as *ω_p_* and *λ_p_*; (3) The frequency corresponding to *ε_r_*=0 in the dielectric real part spectrum is taken as the plasma frequency *ω_p_* to obtain *λ_p_*. Due to the parameters of method (1) are mainly obtained by electrical methods such as a Hall effect tester, there is a certain difference between the calculated *λ_p_'* and the *λ_p_* obtained by optical methods we need. The larger error of method (2) is attributed to the difficulty in determining the location of the maximum slope of the reflection spectrum. In contrast, method (3) is an optical test with a small error, and is a method commonly used in the literature^12^.

When measuring the plasma reflection edge, we use the spectrum of the real part of the dielectric function to obtain plasma frequency *ω_p_* at *ε_r_*=0. We then calculate *λ_p_*, based on *λ_p_*=2πc/*ω_p_*. This method is an optical test with small data error and is commonly used in literature. We obtain plasma reflection edges of Bi_2_Se_2.4_, Bi_2_Te_2.4_, Bi_2_Se_1.8_Te_0.7_ and PbSe_1.6_ using this method, which are 21.4 μm, 18.1 μm, 17.8 μm and 16.5 μm, respectively. These results are in good agreement with data in the literature. Therefore, the *λ_p_* we obtain is accurate^13-16^.

*B7 Electrical and optical properties of thin films with different thicknesses*

To study the effect of thickness on the electrical and optical properties of thin films, we prepared thin film samples with different thicknesses. Fig. S10 and Fig. S11 show the average transmittance spectra (including ZnS substrate) and electrical conductivity of r-Bi_2_Se_2.4_ and r-Bi_2_Se_1.8_Te_0.7_ films with thicknesses of 20-160 nm in the 8-12 μm band, respectively. The r-Bi_2_Se_2.4_ with thicknesses of 20 nm, 40 nm, 80 nm and 160 nm have electrical conductivities of 914 S/cm, 1049 S/cm, 1417 S/cm and 1501 S/cm, respectively. The average transmittance (self-standing film) in the 8-12 μm band are 97.3%, 98.1%, 92.5% and 82.4%, respectively. The electrical conductivities of r-Bi_2_Se_1.8_Te_0.7_ with thicknesses of 20 nm, 40 nm, 80 nm and 160 nm were 923 S/cm, 1460 S/cm, 1928 S/cm and 2453 S/cm, respectively. The average transmittances in the 8-12 μm band were 97.4%, 92.3%, 82.8% and 68.5%, respectively. These results show that the optoelectronic properties of all samples meet the application standards of far-infrared transparent conductive films (transmittance > 70%; conductivity > 1000 S/cm).

*B8 Reasons for the high relaxation time of r-Bi_2_Se_2.4_ films*

The *τ* (7.8 E-11 s) of our prepared r-Bi_2_Se_2.4_ film is greater than that of the ITO film (*τ*=1.1 E-11 s). This great *τ* is mainly attributed to the following two reasons. The first is that the r-Bi_2_Se_3_ crystal has an intrinsic mobility much higher than that of In_2_O_3_:Sn, which can be attributed to the smaller effective mass and greater deformation potential (*E_d_*) of r-Bi_2_Se_3_, , this represents the variation of the valence band maximum (VBM) and conduction band minimum (CBM) with respect to the vacuum energy level under different strains. Similar conclusions have been reported in related studies^17^. Second, because the electron concentration of r-Bi_2_Se_2.4_ thin film is lower than that of ITO thin film, the low electron concentration reduces the collision between electrons, thereby increasing the relaxation time.

*B9 Preparation methods, electrical and optical properties of various transparent conductive materials*

In Fig. 1c, the materials and preparation methods are given, among them, -Epi., CVD, -MBE, -Sputt., -SOG, UT Depo., -PAD represent epitaxy, chemical-vapor deposition, molecular-beam epitaxy, Sputtering, Spin on glass coating, Ultrasonic deposition, Polymer-assisted deposition, respectively. We also summarized several important physical quantities of our prepared Bi_2_Se_2.4_, Bi2Te_2.4_, Bi_2_Se_1.8_Te_0.7_, PbSe_1.6_ and In_1.7_O_3_:Sn_0.3_ films and other transparent conductive film materials in Table S4. They are thickness(*D*), room-temperature conductivity (*σ*), carrier concentration (*n*), relaxation time (*τ*), effective mass (*m**), optical dielectric constant (*ε_opt_*) and free carrier reflection edge (*λ_p_*). In addition, the preparation process and crystal structure are also summarized. Except for the data of material prepared by us, the data of other materials were all from the literature. To ensure the accuracy, the data in the table were obtained from the samples in the same literature.

*B10 ε_opt_ of materials*

Except for our prepared materials, the *ε*_opt_ of other materials were from the “Material Project” database (<https://materialsproject.org/>). In this database, *ε*_poly_^∞^ is defined as the dielectric constant of a polycrystalline sample with electron polarization as the main contribution. This is consistent with the physical meaning of *ε*_opt_ in this paper. Therefore, we took *ε*_poly_^∞^ in the database as *ε*_opt_.

*B11 ε_opt_ of ionic, metal, covalent bonds and electron-deficiency multicenter bonds.*

*ε_opt_* is mainly dependent on the electronic displacement polarization under the high-frequency electric field. In metallic crystals, *ε_opt_* mainly dependent on the displacement polarization of electrons. The metal in electromagnetic waves can be approximately regarded as a plasma composed of ions and free electrons. Therefore, *ε_opt_* approximates 1 as *ω* approaches infinity according to the formula *ε_opt_*=1-(*ω_p_*/*ω*)^2^. In ionic crystals and covalent crystals, *ε_opt_* is mainly dependent on the displacement polarization of bound electrons. Most valence electrons are localized near ions or atoms and are difficult to move, which leads to a low *ε_opt_*. However, in electron-deficiency multicenter bonds crystals, valence electrons are shared by more atoms, resulting in a greater degree of electron delocalization. High electron delocalization increases electron displacement polarization, which in turn leads to a high *ε_opt_*.

**C Data analysis and modeling**

*C1 Electron polarizability calculation*

We calculated the polarizability of the films by the Clausius-Mossotti equation^18^. This equation is :

$\frac{\varepsilon_{\text{opt}}-1}{\varepsilon_{\text{opt}}+2}=\frac{N\alpha_{\text{e}}}{3\varepsilon_{0}}$ (5)

where *n_0_* is the number of atoms per unit volume, *α_e_* is the electron displacement polarization, *ε*_opt_ is the optical frequency permittivity, and *ε*_0_ is the vacuum dielectric constant. The polarizabilities of amorphous and crystalline bismuth selenide films are 6.1 and 36.9, respectively.

*C2 Calculation of polarizability under the 8-N rule*

The polarizability of the bismuth selenide thin films with coordination structures conforming to the 8-N rule was calculated using the asymmetric diatomic molecular model proposed by Pitkonen^19^. Asymmetric diatomic molecules are shown in Fig. S12. The formulae of the model are

$\alpha_{e\parallel}=\frac{1+2(r_{A}r^{-1})^{3}}{1-4(r_{A}r^{-1})^{3}(r_{B}r^{-1})^{3}}\alpha_{B}+\frac{1+2(r_{B}r^{-1})^{3}}{1-4(r_{A}r^{-1})^{3}(r_{B}r^{-1})^{3}}\alpha_{A}$ (6)

$\alpha_{e\perp}=\frac{1-(r_{A}r^{-1})^{3}}{1-(r_{A}r^{-1})^{3}(r_{B}r^{-1})^{3}}\alpha_{B}+\frac{1-(r_{B}r^{-1})^{3}}{1-(r_{A}r^{-1})^{3}(r_{B}r^{-1})^{3}}\alpha_{A}$ (7)

$\overline{\alpha_{e}}=\alpha_{e\perp}+\frac{\alpha_{e\parallel}-\alpha_{e\perp}}{3}$ (8)

where *α*_e∥_ is the polarizability parallel to the electric field direction, *α*_e⊥_ is the polarizability perpendicular to the electric field direction, ‾*α*_e_ is the average polarizability at any position in the electric field after considering the anisotropy. *r*_A_ is the atomic radius of A atom in compound AB, *r*_B_ is the atomic radius of B atom in compound AB, and *r* is the distance between A and B atoms in compound AB. α_A_ is the atomic polarizability of the A atom, and α_B_ is the atomic polarizability of the B atom. The polarizability of the bismuth selenide film whose coordination structure conforms to the 8-N rule is 7.53 E-40 Fm^2^;

*C3 Bohr radius calculation*

Under the action of an external electric field, the electron cloud around the atom is displaced relative to the nucleus. The resulting polarization is called electron displacement polarization. It is an elastic and instantaneous polarization. We used different theoretical models to estimate the value of the electron polarizability *α*_e_. In Fig. S13, atoms are dealt with through Bohr's atomic model. This indicates that a point charge (-*e*) orbits in a circle centered on charge *+q*. The electric field *E* perpendicular to the orbital plane moves +q along the axis from the orbital center to the point M, then the atom-induced dipole moment *μ* is *μ*=*ed*, where *d*=|OM| Then, we have

$\frac{d}{R_{d}}=\frac{eE}{F_{R}}$ (9)

Where *R_b_* is the radius of the electron orbital in Bohr’s atomic model. *F_R_* is the centrifugal force of the homogeneous electron along the orbit. Before the electric field is applied, the attractive force between the nucleus and the electron and the centrifugal force are balanced to form a stable orbit. Then, we have

$\frac{d}{R_{b}}=\frac{eE}{F_{R}}=\frac{eE}{e^{2}/4\pi\varepsilon_{0}R_{b}^{2}}$ (10)

It can be simplified as

$\frac{d}{R_{b}}=\frac{4\pi\varepsilon_{0}R_{b}^{2}E}{e}$ (11)

$\alpha_{e}=\frac{\mu}{E}=\frac{ed}{E}$ is substituted, we have

$\alpha_{e}=4\pi\varepsilon_{0}R_{b}^{3}$ (12)

In Equation (12), the value of the electron polarizability has a great relationship with the Bohr radius.

We calculated the Bohr radius *R_b_* of the bismuth selenide thin film before and after annealing by ^20^. The calculated results show that the Bohr radius of the amorphous and crystalline bismuth selenide thin films are 127 pm and 210 pm, respectively.

*C4 Band gap measurement*

The optical band gap of the thin film was obtained by Tauc fitting of visible-NIR transmission spectra. The absorption coefficient *α* was calculated from the experimental measured values of *T* according to the following approximate relation *α*=-*LnT*/*d*, where *d* is the thickness of the films. We estimated the optical band gap from the absorption coefficient data as a function of wavelength using the Tauc relation (*αhv*)=*B*(*hv-E_g_*)*^n^*, where h*ν* is the photon energy, *α* is the absorption coefficient, *E_g_* is the optical gap, *B* is the band tailing parameter, and *n* = 2 for direct and indirect band gaps, respectively. The band gaps of Bi_2_Se_2.4_, Bi_2_Te_2.4_, Bi_2_Se_1.8_Te_0.7_ and PbSe_1.6_ films are 0.55, 0.56, 0.46 and 0.91, respectively. This is consistent with the results in the literatures^3,16^, and in good agreement with our spectral fitting results.

*C5 Transmission band evaluation*

According to the methods that have been reported^21^, we took 1.24/*E_g_* and *λ_p_* as the transmission cutoff for short wavelength and long wavelength, respectively. According to the *E_g_* and *λ_p_* obtained by our test, the transparent bands of Bi_2_Se_2.4_, Bi_2_Te_2.4_, Bi_2_Se_1.8_Te_0.7_, and PbSe_1.6_ films are 2.3-21.4 μm, 2.3-18.1 μm, 2.7-17.8 μm and 1.4-16.5 μm, respectively.

*C6 The degree of ionization, degree of hybridization and degree of saturation of various materials*

We calculated the degree of ionization and the degree of hybridization of all candidate materials using the method proposed by Wuttig^22^. The data are listed in Table S5. Then, the atomic radius of the calculation results was normalized^23^, and the calculation results are also shown in Table S5. The formulae are

$D_{i}=\left[\left(r_{s}^{M}+r_{p}^{M}+r_{d}^{M}\right)+\left(r_{s}^{N}+r_{p}^{N}+r_{d}^{N}\right)\right]/\frac{r_{M}+r_{N}}{2}$ (13)

$D_{\text{h}}=\left[\left(r_{d}^{M}-r_{s}^{M}\right)+\left(r_{d}^{N}-r_{s}^{N}\right)\right]^{-1}/\frac{r_{M}+r_{N}}{2}$ (14)

$D_{s}=\frac{CoN_{(8-N\text{o}.)rule}}{CoN_{actual}}$ (15)

where *D*_i_ represents the degree of ionization of the material, *D*_h_ represents the degree of hybridization of the material, and *D*_S_ represents the degree of saturation of the material. *r*M s, *r*M p, *r*M d and *r*N s, *r*N p, *r*N d are the *s*, *p*, *d* orbital radii of the cation and anion in compound MN, respectively. *r_M_* is the covalent radius of the cation, and *r_N_* is the covalent radius of the anion. *CoN(*_8-_*_No._*_)rule_ is the coordination number of cations in the crystal structure conforming to the 8-*No.* rule (*No.* represents the number of valence electrons of the M element), and *CoN_actual_* is the coordination number of cations in the actual crystal structure. In low-saturation electron-deficiency multicenter bond (EDMB) crystals, the actual coordination number of central atoms far exceeds the number specified by the 8-*N* rule. In this case, a single valence electron is shared by more atoms, which greatly enhances the delocalization of valence electrons. High electron delocalization increases the electron displacement polarization and optical dielectric constant, which leads to an improvement in the infrared transparent conductive properties of the film.

**D Density functional theory calculations for electron density difference and ELF**

The electron density difference and electron local function (ELF) were obtained by calculations^24^. ELF is defined as:

$ELF=\left[1+\left\{\frac{K(r)}{K_{h}[\rho(r)]}\right\}^{2}\right]^{-1}$ (16)

where *K* is the curvature of the electron pair density, *ρ*(r) is the electron density, and *K*_h_ is the *K* value of the electron gas with density *ρ*. The ELF values in this paper were calculated for different Bi-Se bonding in Bi_8_Se_12_ primary cells. ELF values range from 0 (completely delocalized electrons) to 1 (completely localized electrons), and the associated data are shown in Fig 3. The electron density difference: *Δρ=ρ*_Bi2Se3_*-ρ*_Bi_*-ρ*_Se_, where *ρ*_Bi2Se3_*，ρ*_Bi_*，ρ*_Se_ denote the electron density of Bi_2_Se_3_, Bi and Se, respectively.

**E Electromagnetic shielding performance evaluation**

*E1 Test method for electromagnetic shielding efficiency*

In Fig. S14, we set up an electromagnetic shielding test system with a vector network analyzer as the core. The sample is placed in a sample stage during the test that is isolated from external electromagnetic interference. The two ends of the sample stage are integrated with electromagnetic signal transmitting and receiving devices, respectively. By mathematical modeling and analysis of the original transmitted signal and the received signal after passing through the film, we measured the shielding performances of the film sample against electromagnetic waves in different frequency bands. We first calibrated the instrument under the test environment. After zero calibration of the electromagnetic environment was obtained, the sample was placed in the sample stage. The docking of each hole was checked whether it was in place. After the airtightness of the sample stage was checked, the written test program in the software was called to obtain the noise reduction ability of the film sample to electromagnetic waves in this frequency band. By replacing the sample stage with other sizes and repeating the above operations, we obtained the electromagnetic shielding efficiency of the thin film samples in other frequency bands. Each sample was tested multiple times in various frequency bands to ensure the accuracy of the test results.

To compare the electromagnetic shielding ability of the bismuth selenide film and that of other electromagnetic shielding films, we converted the electromagnetic shielding effectiveness (*SE*(dB)) output by the vector network analyzer into the more intuitive electromagnetic shielding efficiency (*SE*(%)). The physical meaning of the latter is the percentage of the signal intensity of a beam of electromagnetic waves after passing through the shielding film compared to the attenuation before passing through. The conversion formula between the two is

$SE(\%)=1-\left(\frac{1}{10^{\frac{SE(dB)}{10}}}\right)\times100\%$ (17)

*E2 Sample preparation and electromagnetic shielding efficiency test*

The international standard WR62 was referred to meet the test requirements of the Ku-band (12-18 GHz). The thin film samples to be tested were prepared on polysilicon with a cross-sectional area of 15.79*7.89 mm^-2^. The role of the silicon wafer substrate has been deducted during the test.

*E3 Difference between continuous-film and discontinuous-metal mesh in electromagnetic shielding*

The "continuous film" we describe refers to a geometrically continuous film. The traditional electromagnetic shield is usually a geometrically discontinuous metal grid, which is composed of metal wires with submillimeter spacing. However, the discontinuity of such a grid, when plated on the optical window of the photodetector, can diffract the incident beam. This diffraction effect produces harmful scattering light, which greatly compromises the imaging quality of the optical system. In contrast, our "continuous film" type far-infrared electromagnetic shielder is geometrically continuous, which not only simplifies the preparation process but avoids the problem of order of diffraction.

**G Preparation and annealing of thin films**

Details of preparation, annealing conditions, and thicknesses of all films are shown in Table S6.

**
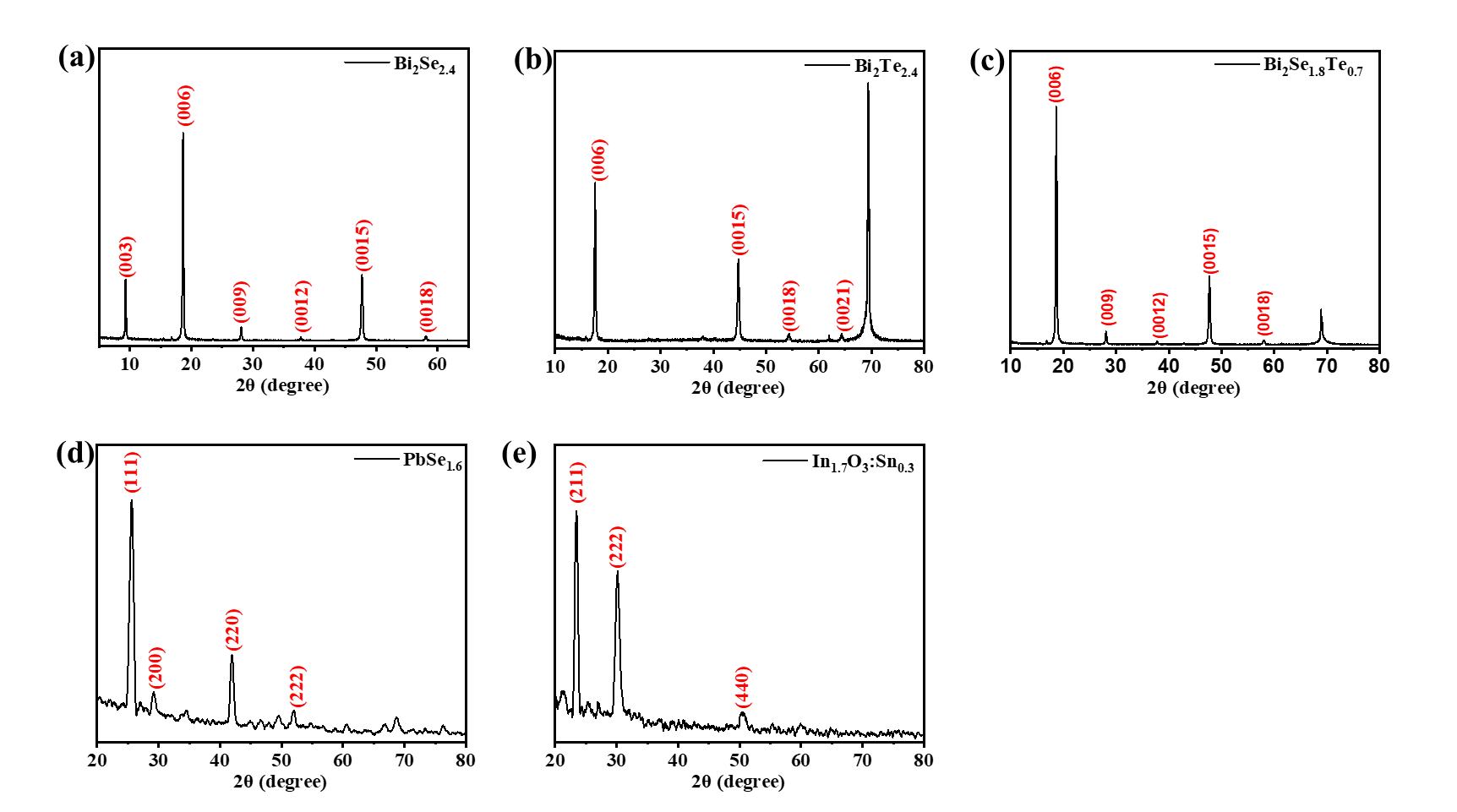
**

**Fig. S1|** GIXRD spectra of (**a**) Bi_2_Se_2.4_, (**b**) Bi_2_Te_2.4_, (**c**) Bi_2_Se_1.8_Te_0.7_, (**d**) PbSe_1.6_ and (**e**) In_1.7_O_3_:Sn_0.3_.


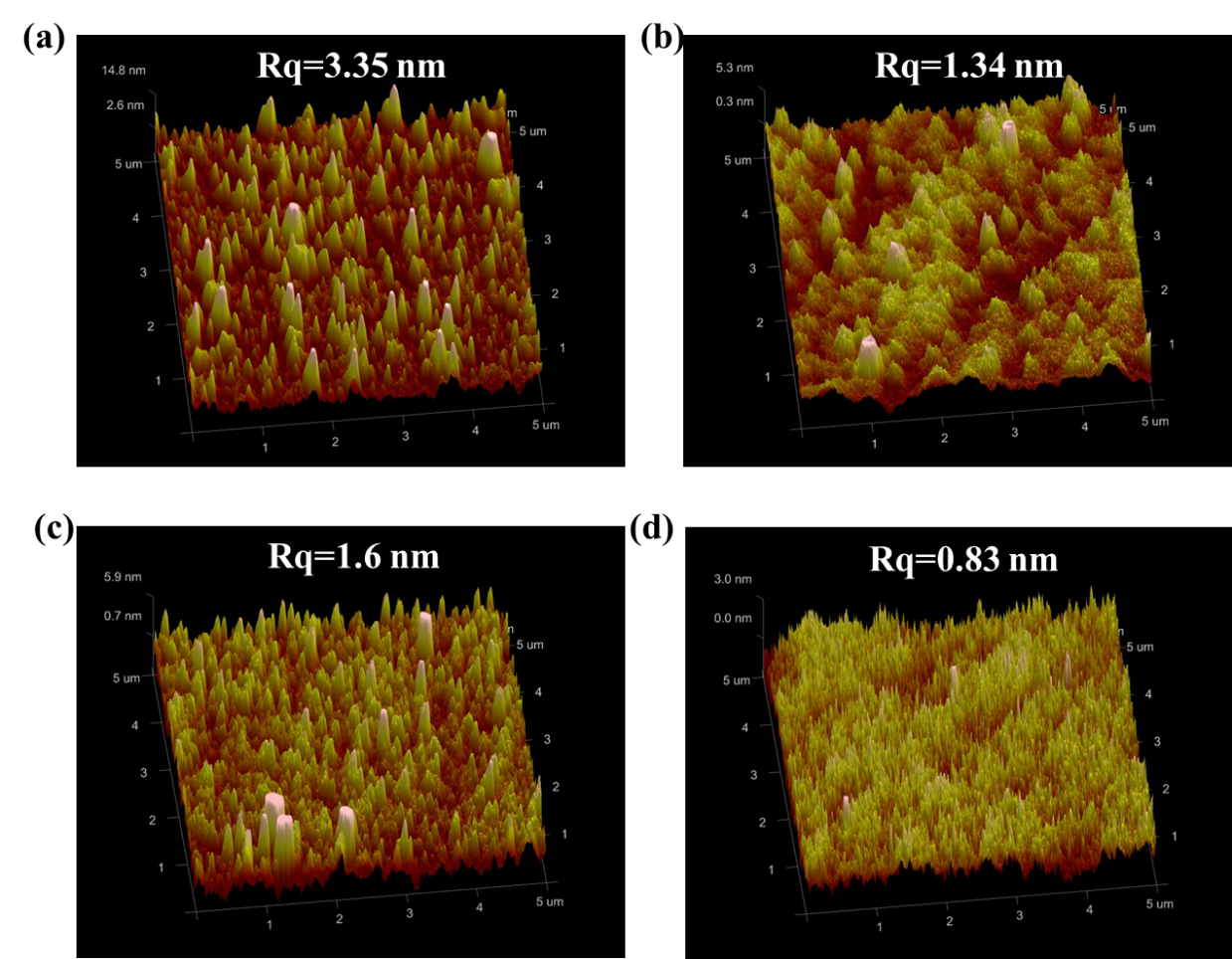


**Fig. S2|** 3D surface morphologies of (**a**) Bi_2_Se_2.4_; (**b**) Bi_2_Te_2.6_; (**c**) Bi_2_Se_1.8_Te_0.7_; and (**d**) PbSe_1.6_ obtained by AFM measurements.

**Fig. S3|** Valence band spectrum of Bi_2_Se_2.4_ film.


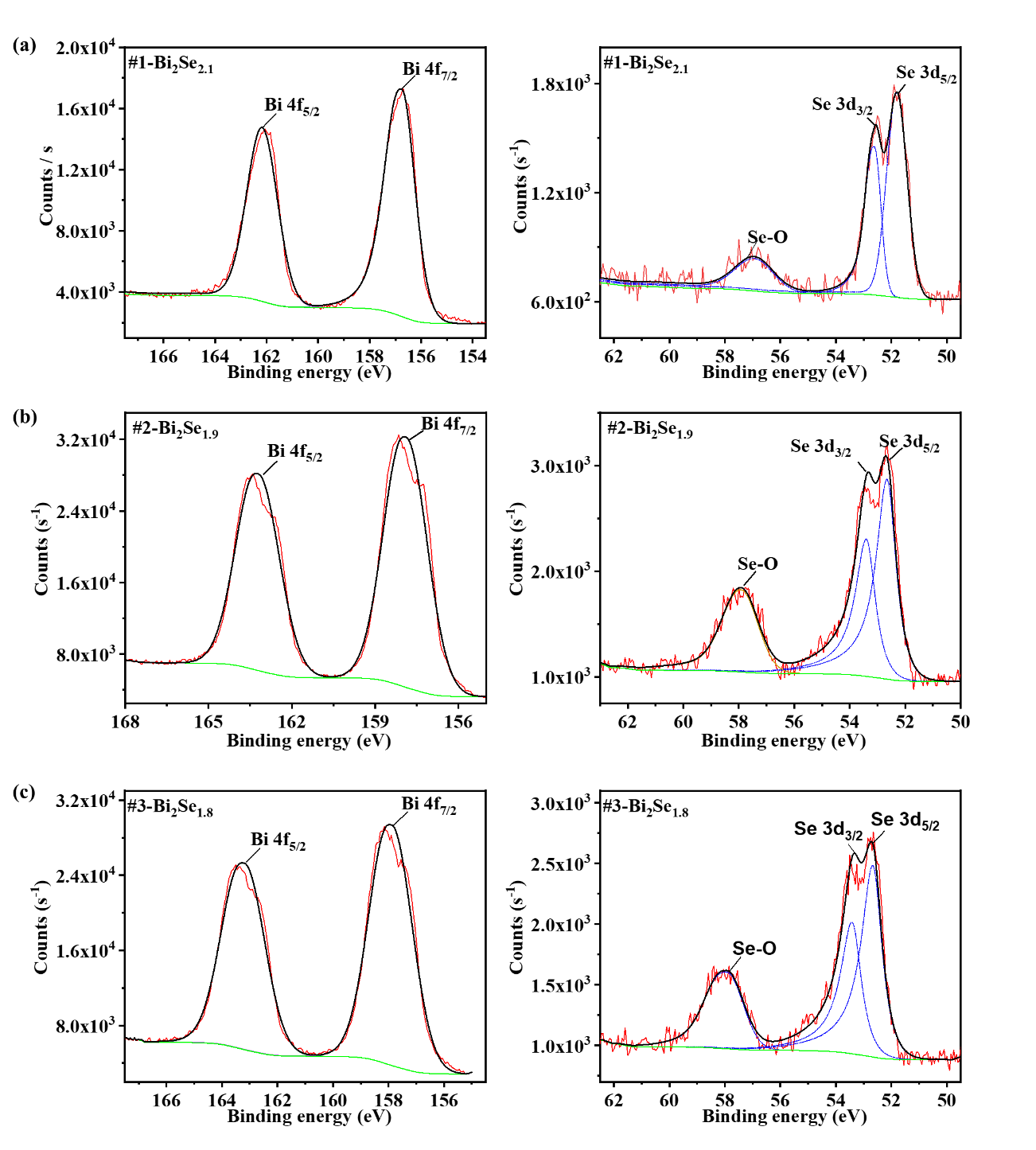


**Fig. S4|** XPS high-resolution elemental scanning spectra of Bi_2_Se_x_ films with annealing temperature of (**a**) 200 ℃, (**b**) 300 ℃, (**c**) 400 ℃ under Ar atmosphere.


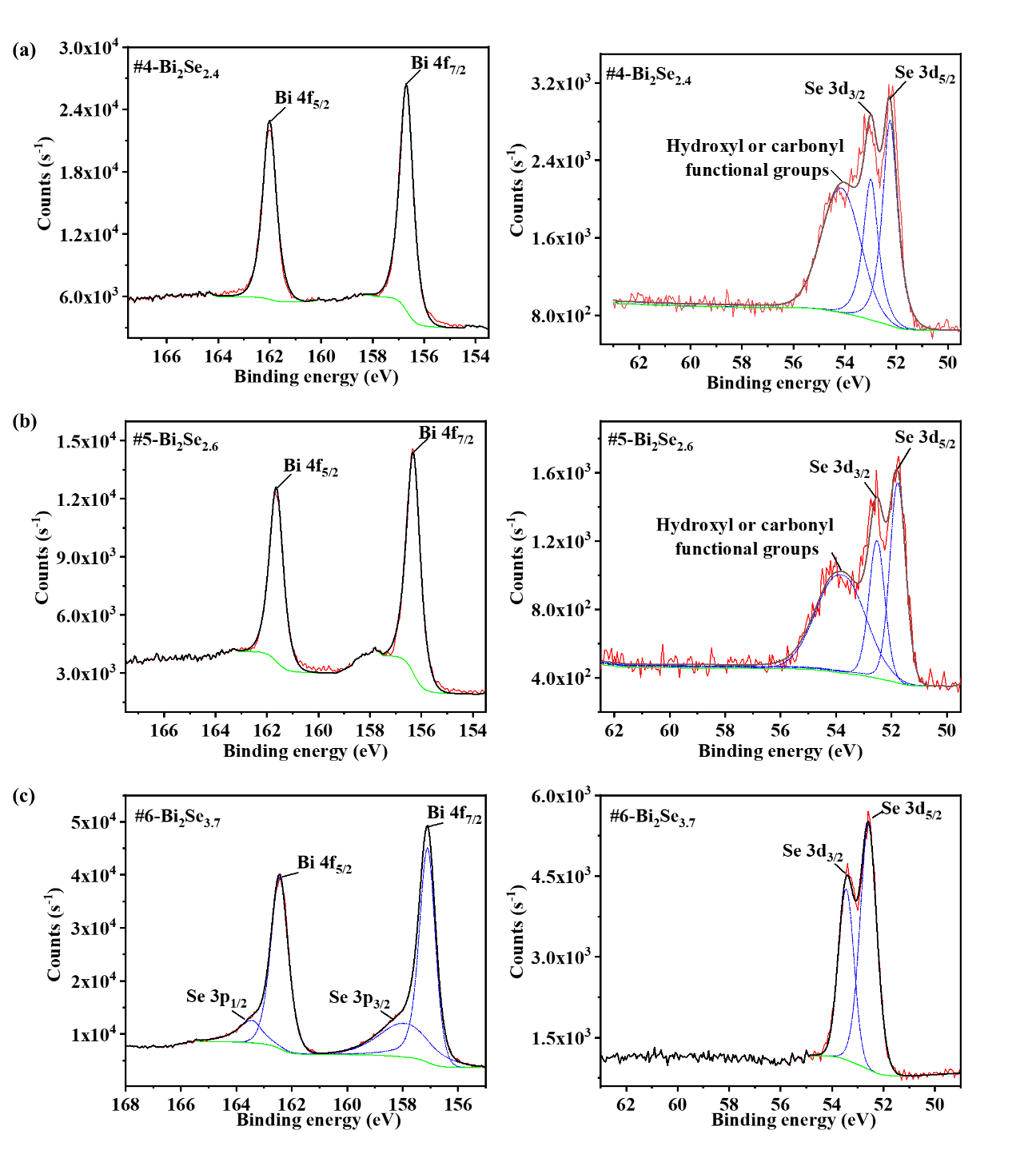


**Fig. S5|** XPS high-resolution elemental scanning spectra of Bi_2_Se_x_ films with annealing temperature of (**a**) 200 ℃, (**b**) 300 ℃, (**c**) 400 ℃ under Se atmosphere.


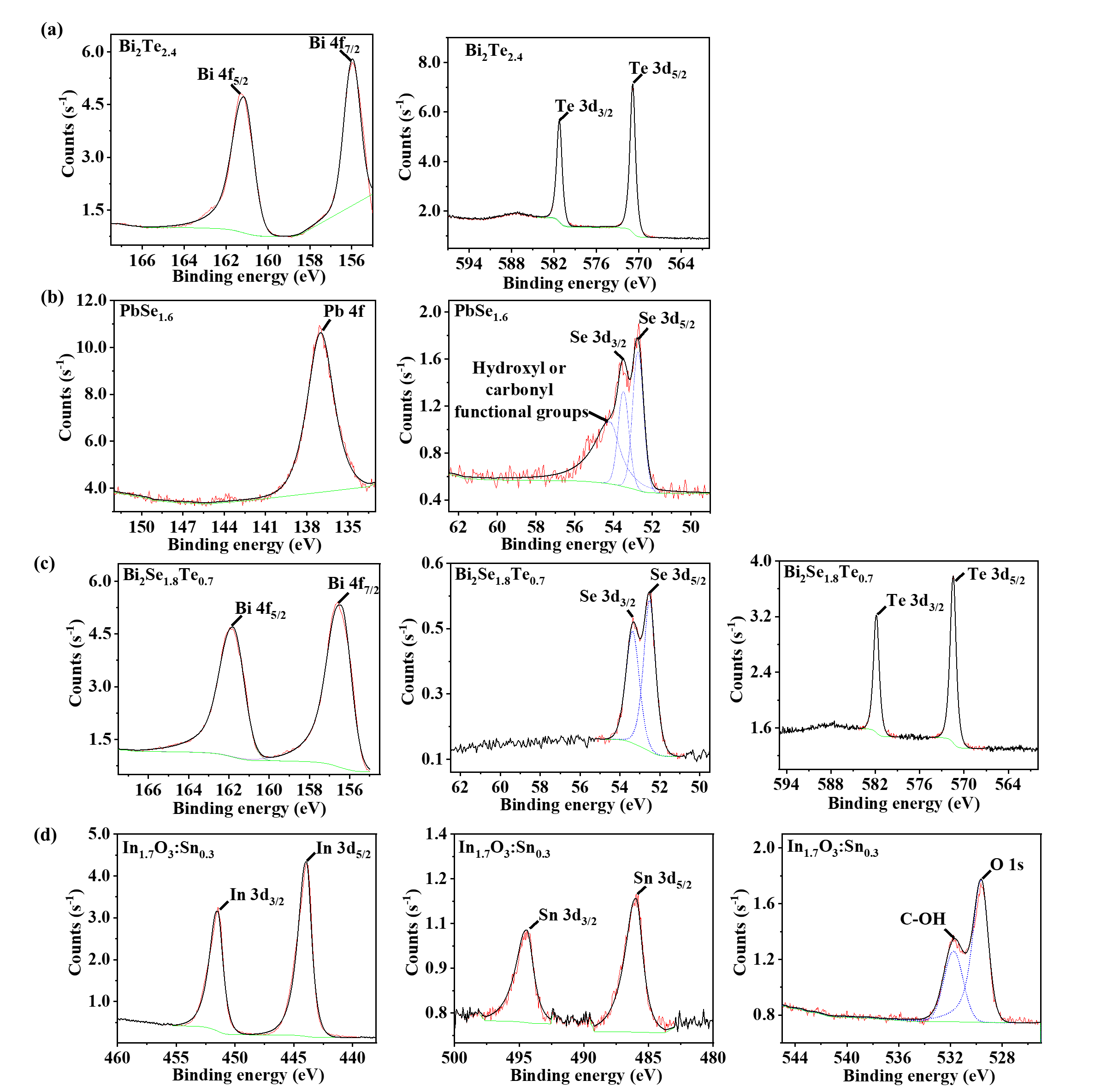


**Fig. S6|** XPS high-resolution spectra of thin films of (**a**) Bi_2_Te_2.4_, (**b**) PbSe_1.6_, (**c**) Bi_2_Se_1.8_Te_0.7_ and (**d**) In_1.7_O_3_:Sn_0.3_.


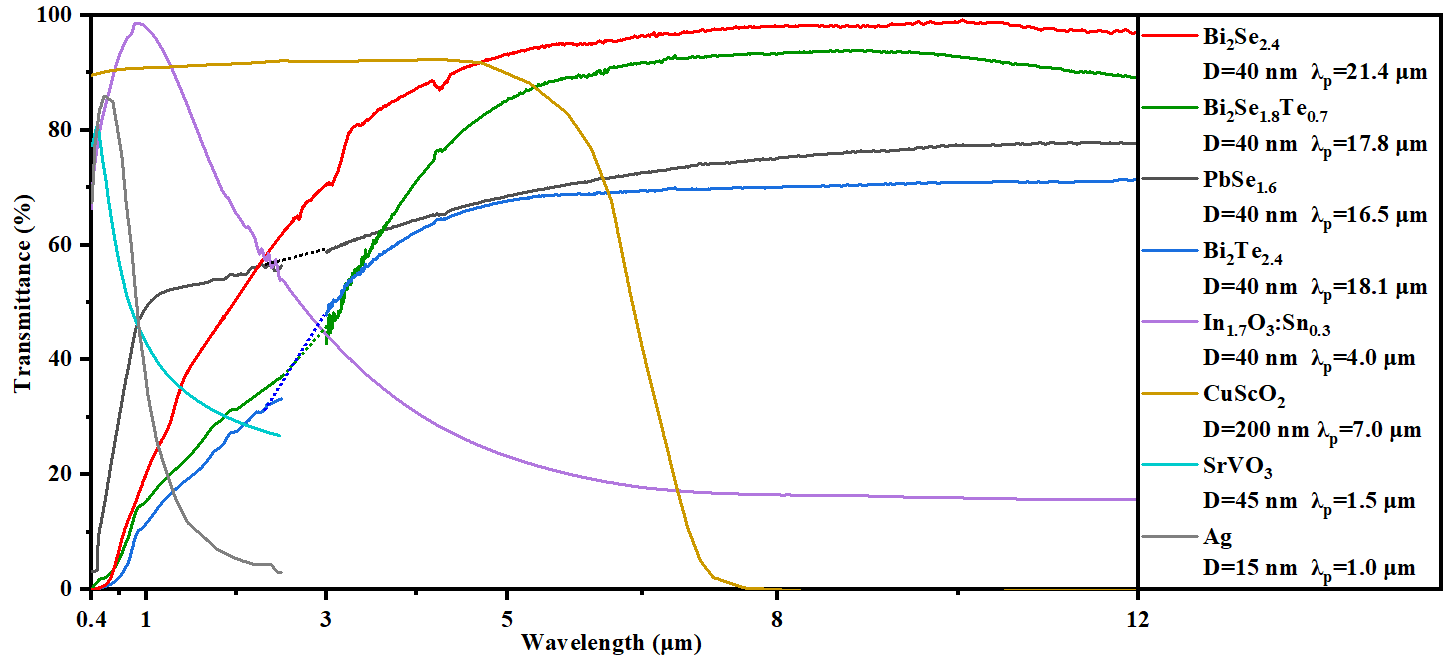


**Fig. S7|** Transmission spectra of self-standing films from visible to far-infrared bands: Bi_2_Se_2.4_, Bi_2_Te_2.4_, Bi_2_Se_1.8_Te_0.7_, PbSe_1.6_, In_1.7_O_3_:Sn_0.3_, Ag, SrVO_3_ and CuScO_2_.


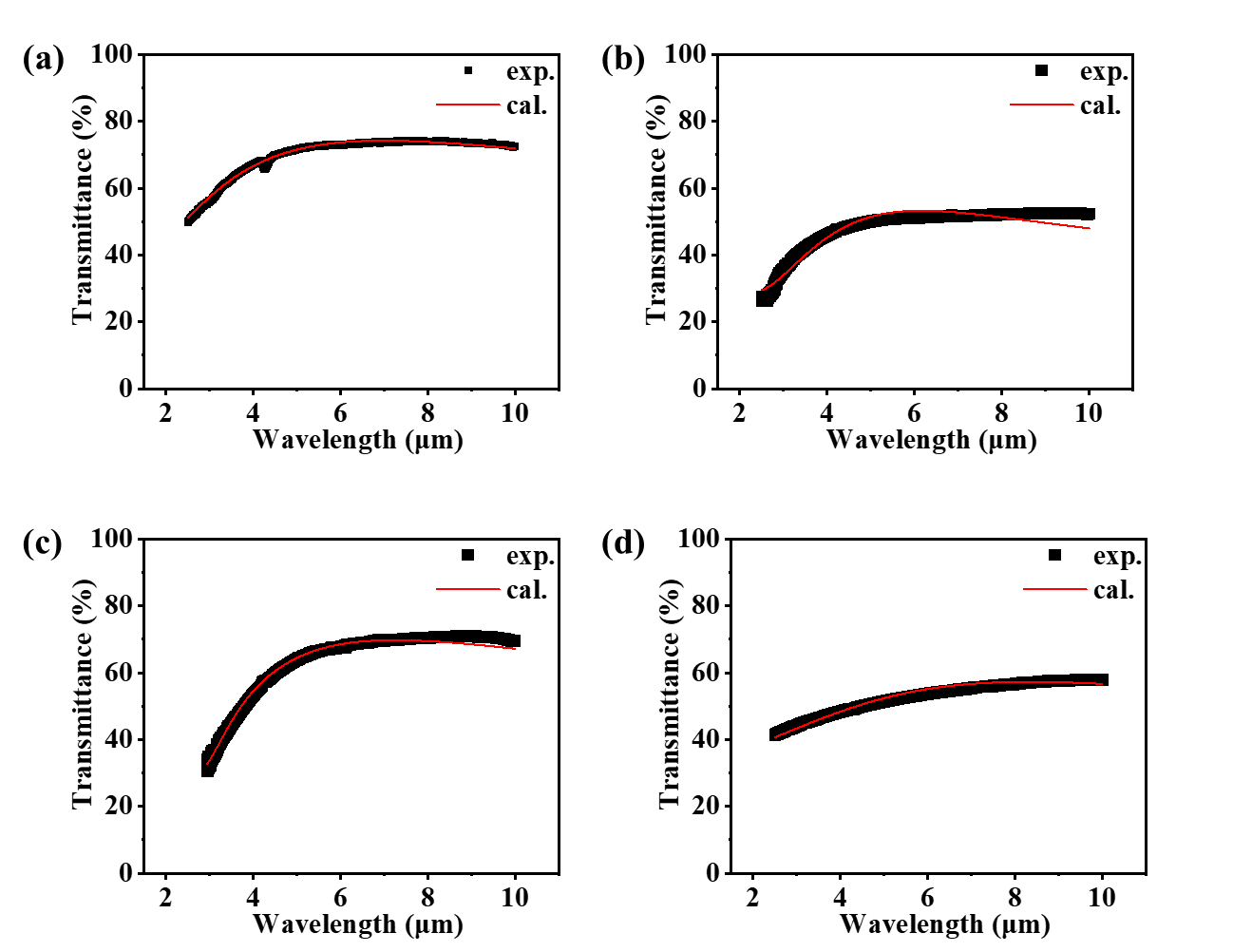


**Fig. S8|** Transmission spectra of (**a**) Bi_2_Se_2.4_, (**b**) Bi_2_Te_2.4_, (**c**) Bi_2_Se_1.8_Te_0.7_ and (**d**) PbSe_1.6_ before and after fitting.


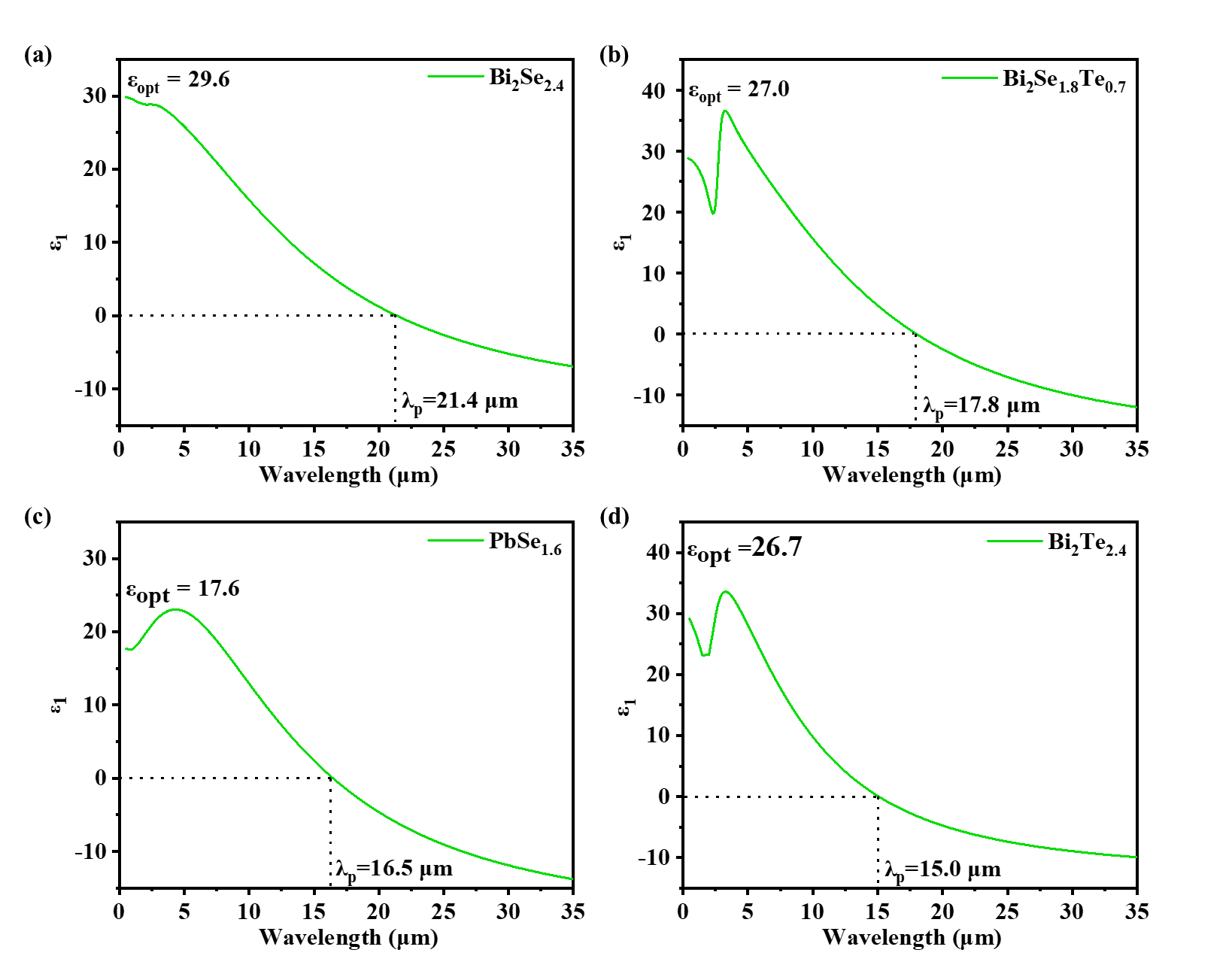


**Fig. S9|** Determination of free carrier reflection edge (*λ*_p_) of films : (a) Bi_2_Se_2.4_, (b) Bi_2_Se_1.8_Te_0.7_, (c) PbSe_1.6_, (d) Bi_2_Te_2.4_.


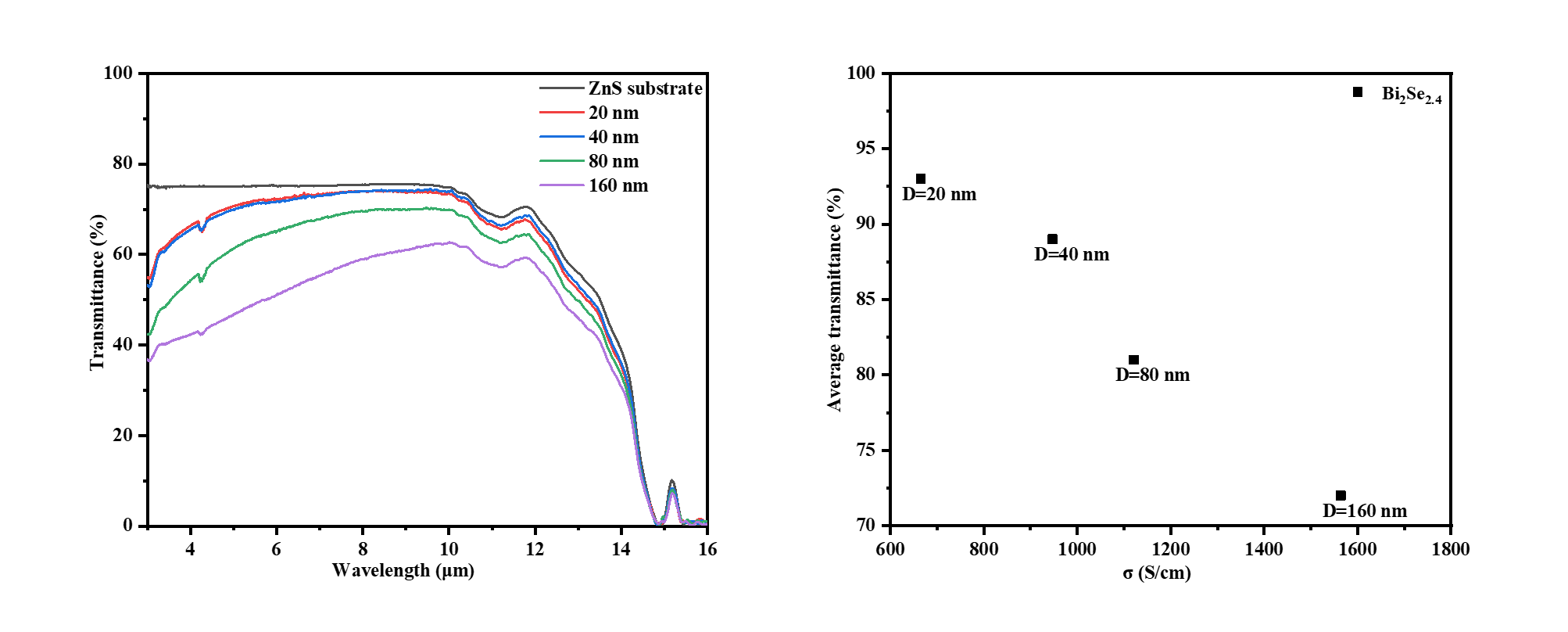


**Fig. S10|** The **(a)** transmission spectra, **(b)** average transmittance (8-12 μm) and electrical conductivity of Bi_2_Se_2.4_ films with different thicknesses (20-160 nm).


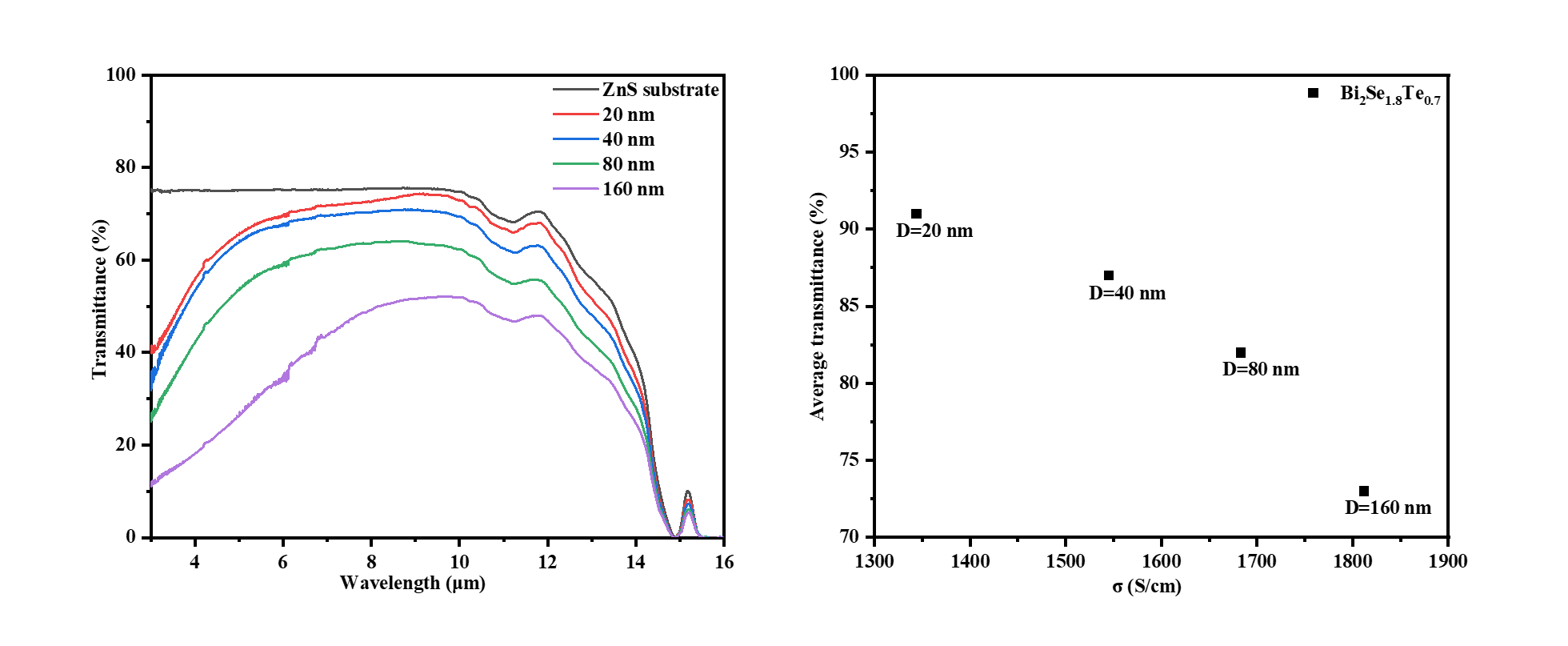


**Fig. S11|** The **(a)** transmission spectra, **(b)** average transmittance (8-12 μm) and electrical conductivity of Bi_2_Se_1.8_Te_0.7_ films at different thicknesses (20-160 nm).


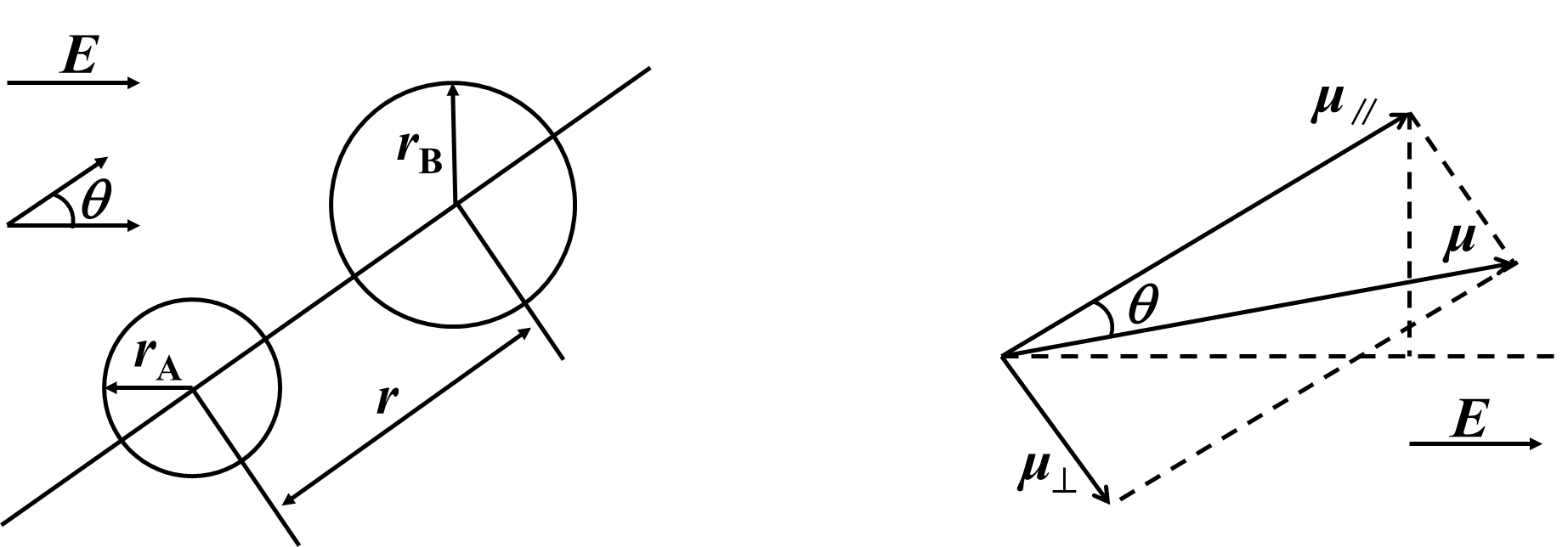


**Fig. S12|** Asymmetric diatomic molecular model.


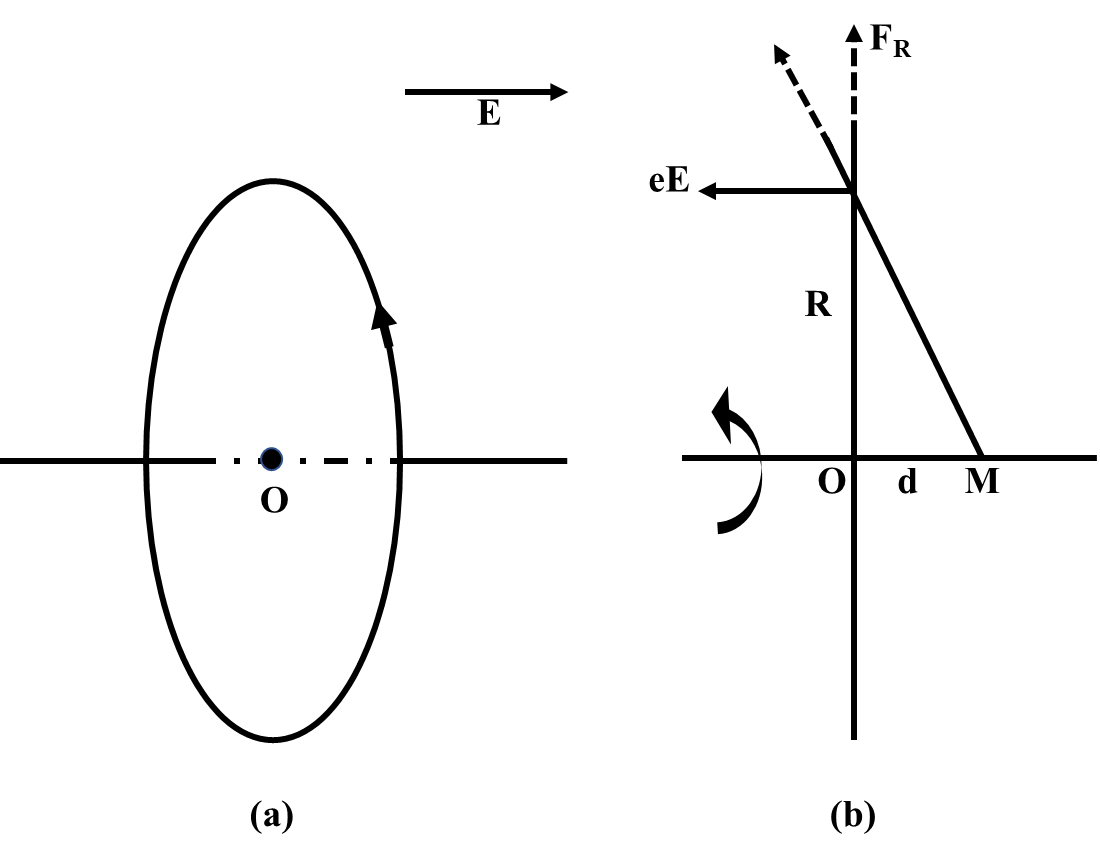


**Fig. S13|** Bohr’s atomic model.


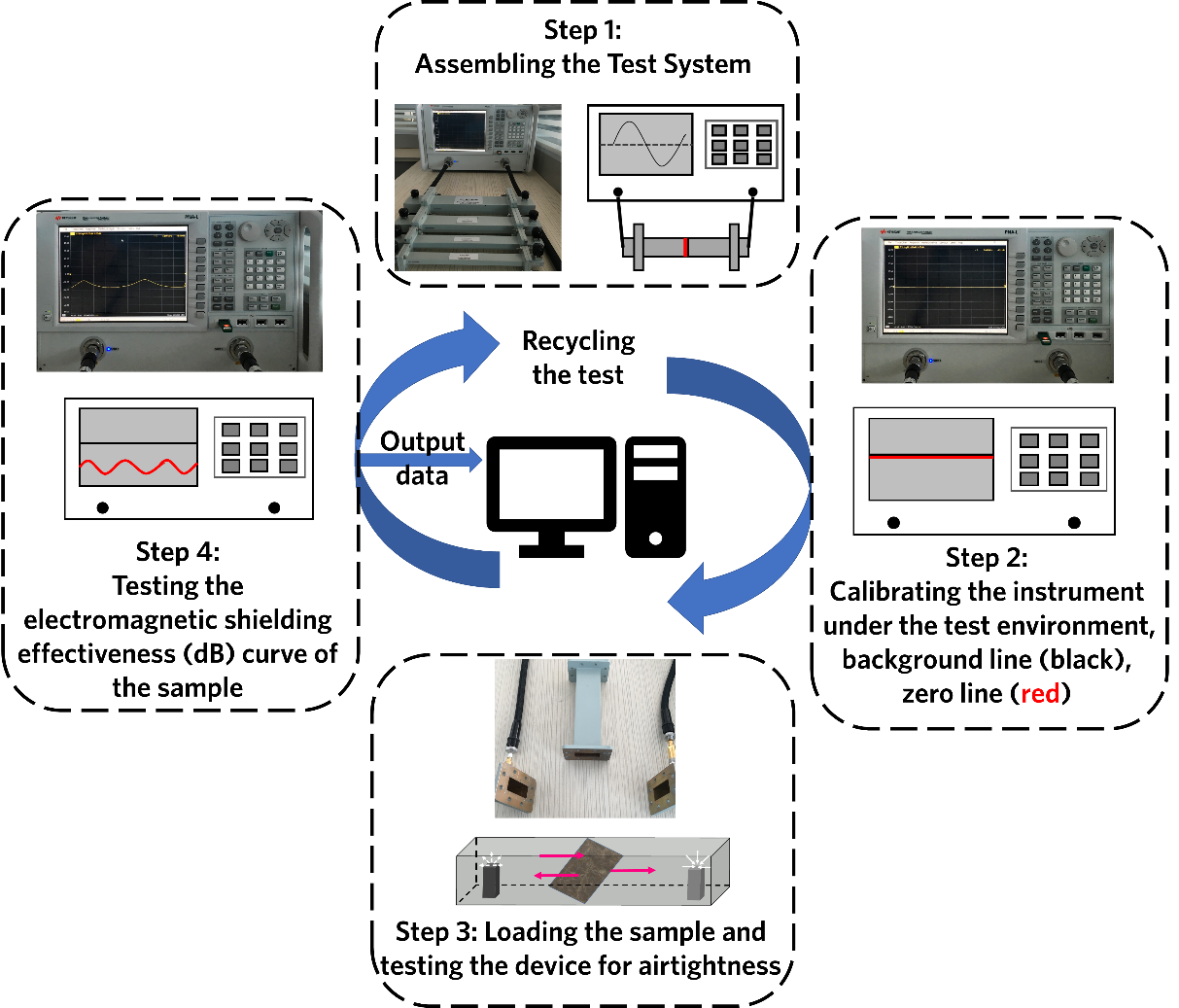


**Fig. S14|** Schematic diagram of electromagnetic shielding test system

**Table S1|** XPS composition analysis results of films after annealing, where *ATM, TEMP* and% represent annealing atmosphere, annealing temperature and element percentage content, respectively.

|  | Annealing  ATM | Annealing  TEMP  ℃ | Bi  % | Pb  % | In  % | Sn  % | Se  % | Te  % | O  % | Stoichiometric ratio |
| --- | --- | --- | --- | --- | --- | --- | --- | --- | --- | --- |
| Bi_x_Se_y_ | As deposited | | 47.1 | NA | | | 52.9 | NA | | 2.0 : 2.3 |
|  | Ar | 200 | 49.3 |  |  |  | 50.7 |  |  | 2.0 : 2.1 |
|  | Ar | 300 | 51.6 |  |  |  | 48.5 |  |  | 2.0 : 1.9 |
|  | Ar | 400 | 53.1 |  |  |  | 47.0 |  |  | 2.0 : 1.8 |
|  | Se | 200 | 45.2 |  |  |  | 54.9 |  |  | 2.0 : 2.4 |
|  | Se | 300 | 43.9 |  |  |  | 56.1 |  |  | 2.0 : 2.6 |
|  | | | | | | | | | | |
| Bi_x_Te_y_ | Te | 300 | 45.5 | NA | | | | 54.5 | NA | 2.0 : 2.4 |
|  | | | | | | | | | | |
| Bi_x_Se_y_Te_z_ | Se,Te | 300 | 45 | NA | | | 39.5 | 15.5 | NA | 2.0 : 1.8 : 0.7 |
|  | | | | | | | | | | |
| PbSe_x_ | Se | 300 | NA | 38.6 | NA | | 61.4 | NA | | 1.0 : 1.6 |
|  | | | | | | | | | | |
| In_x_O_y_:Sn_z_ | Ar | 200 | NA | | 33.1 | 7.2 | NA | | 59.7 | 1.7 : 3.0 : 0.3 |

**Table S2|** Test results of infrared transmittance and Hall electrical characterization, including Hall coefficient (*R*_H_), carrier concentration (*n*), mobility (*μ*), conductivity (*σ*).

|  | *D* | *R*_H_ | *n* | *μ* | *σ* | Average transmittance | | Figure of miret |
| --- | --- | --- | --- | --- | --- | --- | --- | --- |
|  |  |  |  |  |  | 3~5 | 8~12 |  |
|  | nm | cm^3^/C | cm^-3^ | cm^2^/Vs | S/cm | μm | μm | 8~12 μm |
| ZnS-Substrate | NA | | | | | 0.75 | 0.73 | NA |
|  | | | | | | | | |
| Bi_2_Se_2.4_ | 20 | -1.2E-02 | 5.02E+19 | 113.8 | 914 | 0.65 | 0.71 | 5.35E-03 |
|  | 40 | -5.4E-02 | 1.15E+20 | 57 | 1049 | 0.64 | 0.72 | 1.22 E-02 |
|  | 80 | -1.7E-03 | 3.61E+20 | 24.5 | 1417 | 0.53 | 0.68 | 2.89 E-02 |
|  | 160 | -7.8E-03 | 8.01E+20 | 11.7 | 1501 | 0.19 | 0.60 | 4.72 E-02 |
|  | | | | | | | | |
| Bi_2_Se_1.8_Te_0.7_ | 20 | -9.4E-01 | 6.65E+19 | 86.8 | 923 | 0.54 | 0.71 | 5.41 E-03 |
|  | 40 | -5.2E-03 | 1.22E+20 | 70.0 | 1460 | 0.52 | 0.67 | 1.48 E-02 |
|  | 80 | -2.1E-03 | 7.30E+20 | 16.5 | 1928 | 0.41 | 0.60 | 3.06 E-02 |
|  | 160 | -7.7E-02 | 1.30E+21 | 11.8 | 2454 | 0.42 | 0.50 | 5.66 E-02 |
|  | | | | | | | | |
| PbSe_1.6_ | 40 | -4.5E-01 | 1.37E+20 | 30.8 | 674 | 0.48 | 0.56 | 4.67 E-03 |
|  | | | | | | | | |
| Bi_2_Te_2.4_ | 40 | -1.8E-03 | 3.37E+20 | 29.5 | 1589 | 0.45 | 0.52 | 9.42 E-03 |
|  | | | | | | | | |
| In_1.7_O_3_:Sn_0.3_ | 40 | -3.8E-03 | 1.64E+21 | 6.7 | 1763 | 0.47 | 0.23 | 4.81 E-03 |

**Table S3|** Fitting parameters and experimental parameters of films

|  | Oscillator strength | Oscillator broadening | Central energy | ε_∞_ | Fitting | | Experimental | |
| --- | --- | --- | --- | --- | --- | --- | --- | --- |
|  | eV | eV | eV |  | *ω*_p_  eV | E_g_  eV | *ω*_p_  eV | E_g_  eV |
| a-Bi_2_Se_2.4_ | 13.2 | 0.81 | 0.76 | 9 | 1.27 | 0.76 | 1.33 | 0.84 |
| r-Bi_2_Se_2.4_ | 1.1 | 0.5 | 0.56 | 30 | 0.09 | 0.55 | 0.08 | 0.68 |
| Bi_2_Se_1.8_Te_0.7_ | 6.2 | 0.16 | 0.46 | 29 | 0.09 | 0.46 | 0.11 | 0.59 |
| Bi_2_Te_2.4_ | 12.5 | 0.45 | 0.55 | 30 | 0.15 | 0.56 | 0.14 | 0.64 |
| PbSe_1.6_ | 22 | 2.87 | 0.91 | 19 | 0.10 | 0.91 | 0.08 | 0.98 |

**Table S4|** fabrication process, structure, thickness (*D*), conductivity (*σ*), carrier concentration (*n*), relaxation time (*τ*), effective mass (*m**), optical dielectric constant (*ε_opt_*) and free carrier reflection edge (*λ_p_*) of various transparent conductive film materials at room temperature.

| Materials | *D*  nm | *σ*_RT_  S/cm | *n*  cm^-3^ | *τ*  fs | *m*^*^  m_e_ | ε_opt_ | *λ*_p_  μm | Preparation method | Reference |
| --- | --- | --- | --- | --- | --- | --- | --- | --- | --- |
|  |  |  |  |  |  |  |  |  |  |
| SnO_2_ | 446 | 446 | 5.26 E+19 | 89 | 0.3 | 4.7 | 0.7 | Ultrasonic  deposition | [25] |
|  | 600 | 600 | 4.49 E+19 | 141 | 0.3 | 4.7 | 0.8 |  |  |
|  | 716 | 716 | 5.46 E+19 | 138 | 0.3 | 4.7 | 0.7 |  |  |
|  | 788 | 788 | 5.01 E+19 | 166 | 0.3 | 4.7 | 0.8 |  |  |
|  | 696 | 696 | 4.22 E+19 | 174 | 0.3 | 4.7 | 0.8 |  |  |
|  | 707 | 707 | 5.03 E+19 | 148 | 0.3 | 4.7 | 0.8 |  |  |
|  | | | | | | | | | |
| TiO_2_:Nd | 413 | 0.06 | 3.69 E+15 | 247 | 0.4 | 6.8 | 3.9 | Spin-coating | [26] |
|  | 402 | 0.1 | 3.53 E+15 | 465 | 0.4 | 6.8 | 3.7 |  |  |
|  | 395 | 0.09 | 6.07 E+15 | 219 | 0.4 | 6.8 | 2.9 |  |  |
|  | 488 | 3 | 5.56 E+17 | 95 | 0.4 | 6.8 | 3.9 |  |  |
|  | 472 | 4 | 8.31 E+17 | 71 | 0.4 | 6.8 | 3.7 |  |  |
|  | 453 | 5 | 7.89 E+17 | 90 | 0.4 | 6.8 | 3.2 |  |  |
|  | 445 | 7 | 8.34E+17 | 122 | 0.4 | 6.8 | 2.6 |  |  |
|  | | | | | | | | | |
| ZnO:Al | 162 | 1439 | 1.09 E+20 | 125 | 0.3 | 5.4 | 1.7 | Sputtering | [27] |
|  | 180 | 1435 | 1.37 E+20 | 99 | 0.3 | 5.4 | 1.5 |  |  |
|  | 142 | 3636 | 2.60 E+20 | 133 | 0.3 | 5.4 | 1.1 |  |  |
|  | | | | | | | | | |
| ZnO:Al | 152 | 526 | 1.23 E+20 | 41 | 0.3 | 3.8 | 3.9 | Atomic-layer  deposition | [28] |
|  | 151 | 775 | 2.82 E+20 | 26 | 0.3 | 3.8 | 3.8 |  |  |
|  | 153 | 680 | 3.29 E+20 | 20 | 0.3 | 3.8 | 3.5 |  |  |
|  | | | | | | | | | |
| ZnO:Al | 210 | 286 | 2.00 E+20 | 14 | 0.3 | 5.4 | 3 | Sputtering | [29] |
|  | 300 | 399 | 3.20 E+20 | 12 | 0.3 | 5.4 | 2.6 |  |  |
|  | 510 | 645 | 3.00E+20 | 20 | 0.3 | 5.4 | 2.8 |  |  |
|  | 680 | 690 | 2.80 E+20 | 23 | 0.3 | 5.4 | 2.5 |  |  |
|  | 930 | 913 | 4.00 E+20 | 22 | 0.3 | 5.4 | 2.3 |  |  |
|  | | | | | | | | | |
| ZnO:Ga | 60 | 2395 | 6.48 E+20 | 36 | 0.3 | 3.7 | 1.2 | Sputtering | [21] |
| In_2_O_3_:Sn | 69 | 5952 | 1.20 E+21 | 52 | 0.3 | 4.2 | 1 |  |  |
|  | | | | | | | | | |
| In_2_O_3_:Sn | 189 | 1566 | 2.82 E+20 | 62 | 0.3 | 4 | 1.2 | Sputtering | [30] |
|  | 483 | 2000 | 3.77 E+20 | 60 | 0.3 | 4 | 1 |  |  |
|  | 962 | 1930 | 4.14 E+20 | 52 | 0.3 | 4 | 1 |  |  |
|  | | | | | | | | | |
| In_2_O_3_:Sn | 105 | 853 | 3.73 E+20 | 30 | 0.4 | 4 | 3.1 | Sputtering | [31] |
|  | 50 | 487 | 3.45 E+20 | 18 | 0.4 | 4 | 3.4 |  |  |
|  | 15 | 364 | 1.57 E+20 | 30 | 0.4 | 4 | 4.8 |  |  |
|  | | | | | | | | | |
| Materials | *D*  nm | *σ*_RT_  S/cm | *n*  cm^-3^ | *τ*  ps | *m*^*^  m_e_ | ε_opt_ | *λ*_p_  μm | Fabrication process | Reference |
|  |  |  |  |  |  |  |  |  |  |
| ZnO:Al | 150 | 942 | 3.80 E+20 | 24 | 0.3 | 5.4 | 0.7 | Sputtering | [31] |
|  | 142 | 824 | 3.20 E+20 | 24 | 0.3 | 5.4 | 0.8 |  |  |
|  | 120 | 600 | 2.93 E+20 | 19 | 0.3 | 5.4 | 0.8 |  |  |
|  | 120 | 493 | 2.43 E+20 | 19 | 0.3 | 5.4 | 0.9 |  |  |
|  | 120 | 360 | 2.03 E+20 | 17 | 0.3 | 5.4 | 0.9 |  |  |
|  | 75 | 352 | 2.02 E+20 | 17 | 0.3 | 5.4 | 1 |  |  |
|  | | | | | | | | | |
| In_2_O_3_:W | 105 | 2976 | 2.04 E+20 | 138 | 0.3 | 4 | 1.1 | Sputtering | [31] |
|  | 65 | 3135 | 2.01 E+20 | 148 | 0.3 | 4 | 1.1 |  |  |
|  | 65 | 2549 | 2.28 E+20 | 106 | 0.3 | 4 | 1.3 |  |  |
|  | 65 | 1234 | 1.44 E+20 | 81 | 0.3 | 4 | 1.4 |  |  |
|  | | | | | | | | | |
| SrVO_3_ | 4 | 12000 | 2.00 E+22 | 95 | 4.5 | 2.3 | 0.8 | Molecular-beam  epitaxy | [32] |
|  | 10 | 22000 | 2.1 E+22 | 166 | 4.5 | 2.3 | 0.8 |  |  |
|  | 12 | 27000 | 2.10E+22 | 203 | 4.5 | 2.3 | 0.8 |  |  |
|  | 15 | 30000 | 2.40E+22 | 198 | 4.5 | 2.3 | 0.7 |  |  |
|  | 20 | 35000 | 2.60E+22 | 213 | 4.5 | 2.4 | 0.7 |  |  |
|  | 45 | 33000 | 2.25 E+22 | 232 | 4.5 | 2.2 | 0.7 |  |  |
|  | | | | | | | | | |
| CaVO_3_ | 4 | 11000 | 2.25 E+22 | 77 | 4.5 | 2 | 0.7 | Molecular-beam  epitaxy | [32] |
|  | 10 | 17000 | 2.50E+22 | 108 | 4.5 | 2 | 0.7 |  |  |
|  | 14 | 20000 | 2.30E+22 | 138 | 4.5 | 1.9 | 0.7 |  |  |
|  | 18 | 21000 | 2.60E+22 | 128 | 4.5 | 1.9 | 0.6 |  |  |
|  | 38 | 28000 | 2.30E+22 | 193 | 4.5 | 2 | 0.7 |  |  |
|  | | | | | | | | | |
| SrVO_3_ | 30 | 5348 | 2.48 E+22 | 10 | 1.3 | 2.2 | 0.5 | Pulsed-laser  deposition | [33] |
|  | 30 | 5618 | 2.55 E+22 | 14 | 1.8 | 2.2 | 0.5 |  |  |
|  | 30 | 3817 | 2.09 E+22 | 11 | 1.7 | 2.2 | 0.5 |  |  |
|  | 30 | 4831 | 2.26 E+22 | 12 | 1.6 | 2.2 | 0.5 |  |  |
|  | | | | | | | | | |
| CuScO_2_:Sn | 200 | 1 | 1.13 E+18 | 78 | 2.6 | 5.9 | 5.9 | Polymer-assisted  deposition | [34] |
|  | 210 | 5 | 3.40 E+19 | 14 | 2.6 | 5.9 | 4.8 |  |  |
|  | 190 | 18 | 6.20 E+20 | 3 | 2.6 | 5.9 | 5 |  |  |
|  | | | | | | | | | |
| Graphene | 300 | 59.2 | 1.50E+17 | 2808 | 0.2 | NA | 0.57 | Chemical-vapor  deposition | [35] |
|  | 30 | 841.6 | 3.30E+18 | 18155 | 0.2 | NA | 0.47 |  |  |
|  | 30 | 673.7 | 1.90E+18 | 25232 | 0.2 | NA | 0.55 |  |  |
|  | 30 | 952.8 | 3.90E+18 | 17384 | 0.2 | NA | 0.45 |  |  |
|  | | | | | | | | | |
| Ag | / | 64516 | 5.80 E+22 | 12 | 0.3 | 3.3 | 0.2 | NA | [36] |
| Au | / | 45455 | 6.60 E+22 | 7 | 0.3 | 3.4 | 0.2 | NA |  |
|  | | | | | | | | | |
| Cu | 39 | 58823 | 8.45 E+22 | 7 | 0.3 | 3 | 0.6 | NA | [32] |
| Ag | 52 | 62112 | 5.85 E+22 | 11 | 0.3 | 3 | 0.5 | NA |  |
| Al | 15 | 36496 | 1.81 E+23 | 2 | 0.3 | 3 | 0.1 | NA |  |

**Table S5|** The orbital radius, coordination number and calculation results of *D_i_*, *D_h_*, *D_s_*;

|  | *r*M s | *r*M p | *r*M d | *r*N s | *r*N p | *r*N d | *ECoN* | | *D*_i_ | *D*_h_ | *D_s_* |
| --- | --- | --- | --- | --- | --- | --- | --- | --- | --- | --- | --- |
|  |  |  |  |  |  |  | 8-*No.* rule | actual |  |  |  |
| PI_2_ | 0.59 | 0.76 | 1.13 | 0.66 | 0.85 | 1.37 | 3 | 2 | 3.23 E-03 | 6.45E-03 | 1.5 |
| GeI_2_ | 0.64 | 0.92 | 1.68 | 0.66 | 0.85 | 1.37 | 4 | 2 | 2.78 E-03 | 4.42E-03 | 2 |
| SnI_4_ | 0.76 | 1.07 | 1.87 | 0.66 | 0.85 | 1.37 | 4 | 4 | 6.09 E-03 | 4.08E-03 | 1 |
| SbI_3_ | 0.72 | 0.97 | 1.56 | 0.66 | 0.85 | 1.37 | 3 | 3 | 2.76 E-03 | 4.81E-03 | 1 |
| BiI_3_ | 0.72 | 1.02 | 1.78 | 0.66 | 0.85 | 1.37 | 3 | 3 | 4.70 E-03 | 4.15E-03 | 1 |
| PbI_3_ | 0.72 | 1.09 | 1.98 | 0.66 | 0.85 | 1.37 | 4 | 3 | 6.67 E-03 | 3.72E-03 | 1.3 |
| AsI_3_ | 0.62 | 0.83 | 1.41 | 0.66 | 0.85 | 1.37 | 3 | 3 | 1.54 E-04 | 5.15E-03 | 1 |
| GeS_2_ | 0.64 | 0.92 | 1.68 | 0.53 | 0.66 | 0.94 | 4 | 2 | 1.02 E-02 | 6.34E-03 | 2 |
| Ga_2_S_3_ | 0.66 | 1.04 | 2.15 | 0.53 | 0.66 | 0.94 | 5 | 4 | 1.54 E-02 | 4.72E-03 | 1.3 |
| SnS | 0.76 | 1.07 | 1.87 | 0.53 | 0.66 | 0.94 | 4 | 3 | 1.29 E-02 | 5.41E-03 | 1.3 |
| Sb_2_S_3_ | 0.72 | 0.97 | 1.56 | 0.53 | 0.66 | 0.94 | 3 | 3 | 9.62 E-03 | 6.87E-03 | 1 |
| PbS | 0.72 | 1.09 | 1.98 | 0.53 | 0.66 | 0.94 | 4 | 3 | 1.33E-02 | 4.81E-03 | 1.3 |
| Bi_2_S_3_ | 0.72 | 1.02 | 1.78 | 0.53 | 0.66 | 0.94 | 3 | 3 | 1.16 E-02 | 5.69E-03 | 1 |
| P_4_S_5_ | 0.59 | 0.76 | 1.13 | 0.53 | 0.66 | 0.94 | 3 | 3 | 3.38 E-03 | 1.02E-02 | 1 |
| As_2_S_3_ | 0.62 | 0.83 | 1.41 | 0.53 | 0.66 | 0.94 | 3 | 3 | 6.71 E-03 | 7.66E-03 | 1 |
| r-Sb_2_Te_3_ | 0.72 | 0.97 | 1.56 | 0.69 | 0.89 | 1.35 | 3 | 6 | 2.35 E-03 | 4.89E-03 | 0.5 |
| r-As_2_Te_3_ | 0.62 | 0.83 | 1.41 | 0.69 | 0.89 | 1.35 | 3 | 6 | 5.44 E-04 | 5.36E-03 | 0.5 |
| r-As_2_Se_3_ | 0.62 | 0.83 | 1.41 | 0.59 | 0.76 | 1.21 | 2 | 6 | 2.56 E-03 | 6.05E-03 | 0.5 |
| r-Bi_2_TeSe_2_ | 0.72 | 1.02 | 1.78 | 0.62 | 0.81 | 1.26 | 2 | 6 | 6.35 E-03 | 4.48E-03 | 0.5 |
| r-Sb_2_TeSe_2_ | 0.72 | 0.97 | 1.56 | 0.62 | 0.81 | 1.26 | 2 | 6 | 4.41 E-03 | 5.28E-03 | 0.5 |
| r-Sb_2_Te_2_Se | 0.72 | 0.97 | 1.56 | 0.66 | 0.85 | 1.3 | 2 | 6 | 3.35 E-03 | 5.08E-03 | 0.5 |
| r-Bi_2_Te_3_ | 0.72 | 1.02 | 1.78 | 0.69 | 0.89 | 1.35 | 3 | 6 | 4.23 E-03 | 4.17E-03 | 0.5 |
| r-Bi_2_Se_3_ | 0.72 | 1.02 | 1.78 | 0.59 | 0.76 | 1.21 | 2 | 6 | 7.50 E-03 | 4.65E-03 | 0.5 |
| r-Bi_2_Te_2_Se | 0.72 | 1.02 | 1.78 | 0.66 | 0.85 | 1.3 | 2 | 6 | 5.26 E-03 | 4.32E-03 | 0.5 |
| c-PbSe | 0.72 | 1.09 | 1.98 | 0.59 | 0.76 | 1.21 | 4 | 6 | 9.35 E-03 | 4.04E-03 | 0.7 |
| c-GeTe | 0.64 | 0.92 | 1.68 | 0.69 | 0.89 | 1.35 | 4 | 6 | 2.41 E-03 | 4.58E-03 | 0.7 |
| c-SnTe | 0.76 | 1.07 | 1.87 | 0.69 | 0.89 | 1.35 | 4 | 6 | 5.58 E-03 | 4.09E-03 | 0.7 |
| c-PbTe | 0.72 | 1.09 | 1.98 | 0.69 | 0.89 | 1.35 | 4 | 6 | 6.10 E-03 | 3.69E-03 | 0.7 |
| c-SnSe | 0.76 | 1.07 | 1.87 | 0.59 | 0.76 | 1.21 | 4 | 6 | 8.87 E-03 | 4.50E-03 | 0.7 |
| In_2_O_3_ | 0.76 | 1.17 | 2.31 | 0.28 | 0.32 | 1 | 5 | 6 | 2.60 E-02 | 4.34E-03 | 0.8 |
| CdO | 0.71 | 1.32 | 3.18 | 0.28 | 0.32 | 1 | 6 | 6 | 3.27 E-02 | 2.84E-03 | 1 |
| SnO_2_ | 0.76 | 1.07 | 1.87 | 0.28 | 0.32 | 1 | 4 | 6 | 2.20 E-02 | 5.71E-03 | 0.7 |
| CuScO_2_ | 0.43 | 1.61 | 5.89 | 0.28 | 0.32 | 1 | 5 | 6 | 5.92 E-02 | 1.51E-03 | 0.8 |
| CuAlO_2_ | 0.59 | 1.35 | 3.91 | 0.28 | 0.32 | 1 | 5 | 6 | 4.21 E-02 | 2.46E-03 | 0.8 |
| CuGaO_2_ | 0.55 | 1.33 | 4.02 | 0.28 | 0.32 | 1 | 5 | 6 | 4.19 E-02 | 2.33E-03 | 0.8 |
| SrCu_2_O_2_ | 0.74 | 1.77 | 4.5 | 0.28 | 0.32 | 1 | 6 | 6 | 5.28 E-02 | 2.18E-03 | 1 |
| SrTiO_3_ | 1.36 | 2.09 | 1.74 | 0.28 | 0.32 | 1 | 5 | 6 | 3.28 E-02 | 8.31E-03 | 0.8 |
| BaSnO_3_ | 1.16 | 1.71 | 1.79 | 0.28 | 0.32 | 1 | 5 | 6 | 2.73 E-02 | 6.61E-03 | 0.8 |
| SrVO_3_ | 1.36 | 2.09 | 1.74 | 0.28 | 0.32 | 1 | 4.5 | 6 | 3.35 E-02 | 8.48E-03 | 0.8 |
| CaVO_3_ | 1.22 | 1.87 | 1.52 | 0.28 | 0.32 | 1 | 4.5 | 6 | 2.91 E-02 | 9.46E-03 | 0.8 |

**Table S6|** Deposition and annealing parameters of thin films.

|  | Sputtering | | | | Annealing | | |
| --- | --- | --- | --- | --- | --- | --- | --- |
|  | Target material | RF power  W | TEMP  ℃ | Time  s | ATM | TEMP  ℃ | Time  min |
| Bi_2_Se_x_ | Bi_2_Se_3_ | 60 | 300 | 120 | Se | 300 | 15 |
| Bi_2_Te_x_ | Bi_2_Te_3_ | 60 | 300 | 120 | Te | 300 | 15 |
| Bi_2_Se_x_Te_y_ | Bi_2_Se_3_/Bi_2_Te_3_ | 90/60 | 300 | 90 | Se/Te | 300 | 15 |
|  |  |  |  |  |  |  |  |
| PbSe_x_ | PbSe | 80 | 300 | 480 | Se | 300 | 15 |
| In_x_O_y_:Sn_z_ | In_2_O_3_:Sn | 70 | 200 | 300 | Ar | 250 | 15 |

**Table S7|** The differences in preparation, composition, structure, properties, and applications between the indium-tin oxides used commonly and our far-infrared transparent conductive materials. PLD, Sputt. and TE represent Pulsed laser deposition, Sputtering and Thermal evaporation, respectively.

| **Composition**  **& Preparation** | **Struct-ure** | **Properties** | | | | | | **Application** |
| --- | --- | --- | --- | --- | --- | --- | --- | --- |
|  |  | **Optical** | | | **Electrical** | | |  |
|  |  | λ_g_  um | λ_p_  um | T  % | n cm^-3^ | μ cm^2^/Vs | σ S/cm |  |
| In_2_O_3_:Sn^37^  (PLD) | cubic | 0.4 | 1.7 | 90 | 9.00E+20 | 24.2 | 1111 | Flat panel display; Solar cell |
| In_2_O_3_:Sn^38^  (PLD) | cubic | 0.4 | 1.5 | 80 | 8.00E+20 | 26.5 | 3704 |  |
| In_2_O_3_:Sn^39^  (Sputt.) | cubic | 0.4 | 1.3 | 80 | 1.30E+21 | 35.0 | 7143 |  |
| In_2_O_3_:Sn^40^  (Sputt.) | cubic | 0.4 | 1.1 | 80 | 1.00E+21 | 20.0 | 33333 |  |
| In_2_O_3_:Sn^41^  (Sputt.) | cubic | 0.4 | 1.3 | 90 | 8.00E+20 | 29.0 | 3571 |  |
| In_2_O_3_:Sn (this work) | cubic | 0.4 | 1.5 | 90 | 1.60E+21 | 6.7 | 1763 |  |
| Bi_2_Se_2.4_  (Sputt.) | Rhombic | 2.3 | 21.4 | 90 | 1.15E+20 | 57.0 | 1049 | FIR electromagnetic shielding window; FIR photodetector |
| Bi_2_Te_2.4_  (Sputt.) | Rhombic | 2.3 | 18.1 | 60 | 3.37E+20 | 29.5 | 1589 |  |
| Bi_2_Se_1.8_Te_0.7_  (Sputt.) | Rhombic | 2.7 | 17.8 | 90 | 1.22E+20 | 70.0 | 1460 |  |
| PbSe_1.6_  (Sputt.) | cubic | 1.4 | 16.5 | 70 | 1.37E+20 | 30.8 | 674 |  |

**Table S8|** ε_opt_ of ionic, metal, covalent bonds and electron-deficiency multicenter bonds.

| Chemical bond type | Material | Optical dielectric constant (*ε_opt_*) |
| --- | --- | --- |
| Metal | Ag^42^ | 3.3 |
|  | Au^42^ | 3.4 |
| Covalent | C^42^ | 5.8 |
|  | CdS^42^ | 6.4 |
|  | SnO^42^ | 7.3 |
|  | ZnS^42^ | 5.8 |
| Ionic | MgO^43^ | 3.2 |
|  | CaTe^43^ | 6.6 |
|  | NaCl^43^ | 2.5 |
|  | KBr^43^ | 2.5 |
| electron-deficiency  multicenter bonds  (this work) | r-Bi_2_Se_2.4_ | 29.6 |
|  | r-Bi_2_Te_2.4_ | 26.7 |
|  | r-Bi_2_Te_1.8_Se_0.7_ | 17.6 |
|  | c-PbSe_1.6_ | 27.0 |

The table shows that ITO is more suitable for applications in the visible to near-infrared band, whereas the films we prepare (e. g. bismuth selenide) are more suitable for applications in the mid to far infrared.

**References**

1 Huang Fei *et al.* Raman Spectra of Bi_2_Se_3_ Single Crystals at Different Laser Wavelengths and Powers. *J Chin Ceram Soc* **47**, 514-518, (2019).

2 Fülöp, A. *et al.* Phase transition of bismuth telluride thin films grown by MBE. *Appl. Phys. Express* **7**, (2014).

3 Dubroka, A. *et al.* Interband absorption edge in the topological insulators Bi_2_(Te_1−x_Se_x_)_3_. *Phys. Rev. B* **96**, (2017).

4 Preetha, K. C. & Remadevi, T. L. Band gap engineering in PbSe thin films from near-infrared to visible region by photochemical deposition method. *J. Mater. Sci.-Mater. Electron.* **25**, 1783-1791, (2014).

5 Gong, W. *et al.* Investigation of In_2_O_3_:SnO_2_ films with different doping ratio and application as transparent conducting electrode in silicon heterojunction solar cell. *Sol. Energy Mater. Sol. Cells* **234**, (2022).

6 V.B. Nascimento *et al.* XPS and EELS study of the bismuth selenide. *J. Electron Spectrosc. Relat. Phenom.* **104**, 99-107 (1999).

7 Wang, D. *et al.* Designing hard, low-refractive-index lossy materials for super wear-resistant absorbers. *Mater. Res. Lett.* **10**, 472-480, (2022).

8 Shu, G. J. *et al.* Dynamic surface electronic reconstruction as symmetry-protected topological orders in topological insulator Bi_2_Se_3_. *Phys. Rev. Mater.* **2**, (2018).

9 Mendoza-Galván, A. & González-Hernández, J. Drude-like behavior of Ge:Sb:Te alloys in the infrared. *J. Appl. Phys.* **87**, 760-765, (2000).

10 Gordon, R. G. Criteria for choosing transparent conductors. *MRS Bull.* **25**, 52-57 (2000).

11 Cochran, W. *The Structure and Properties of Solids*. (Edward Arnold, 1973).

12 Lai, F., Lin, L., Gai, R., Lin, Y. & Huang, Z. Determination of optical constants and thicknesses of In_2_O_3_:Sn films from transmittance data. *Thin Solid Films* **515**, 7387-7392, (2007).

13 Koc, H., Ozisik, H., Deligoz, E., Mamedov, A. M. & Ozbay, E. Mechanical, electronic, and optical properties of Bi_2_S_3_ and Bi_2_Se_3_ compounds: first principle investigations. *J. Mol. Model.* **20**, 2180, (2014).

14 Caha, O. *et al.* Growth, structure, and electronic properties of epitaxial bismuth telluride topological insulator films on BaF_2_ (111) substrates. *Cryst. Growth Des.* **13**, (2013).

15 Peiris, F. C. *et al.* Optical properties of Bi_2_(Te_1−x_Se_x_)_3_ thin films. *J. Vac. Sci. Technol. B* **37**, (2019).

16 Allan, G. & Delerue, C. Unusual quantum confinement effects in IV–VI materials. *Mater. Sci. Eng. C* **25**, 687-690, (2005).

17 Liu, Y., Xu, Y., Ji, Y. & Zhang, H. Monolayer Bi_2_Se_3-x_Te_x_: novel two-dimensional semiconductors with excellent stability and high electron mobility. *Phys. Chem. Chem. Phys.* **22**, 9685-9692, (2020).

18 Shportko, K. *et al.* Resonant bonding in crystalline phase-change materials. *Nat. Mater.* **7**, 653-658, (2008).

19 Pitkonen, M. Polarizability of the dielectric double-sphere. *J. Math. Phys.* **47**, (2006).

20 Vegesna, S. V. *et al.* Increased static dielectric constant in ZnMnO and ZnCoO thin films with bound magnetic polarons. *Sci Rep* **10**, 6698, (2020).

21 Fujiwara, H. & Kondo, M. Effects of carrier concentration on the dielectric function of ZnO:Ga andIn_2_O_3_:Snstudied by spectroscopic ellipsometry: Analysis of free-carrier and band-edge absorption. *Phys. Rev. B* **71**, (2005).

22 Lencer, D. *et al.* A map for phase-change materials. *Nat. Mater.* **7**, 972-977, (2008).

23 Chelikowsky, J. R. & Phillips, J. C. Quantum-defect theory of heats of formation and structural transition energies of liquid and solid simple metal alloys and compounds. *Phys. Rev. B* **17**, 2453-2477, (1978).

24 Ptok, A., Kapcia, K. J. & Ciechan, A. Electronic properties of Bi_2_Se_3_ dopped by 3d transition metal (Mn, Fe, Co, or Ni) ions. *J. Phys.-Condes. Matter* **33**, 065501, (2021).

25 Rahal, A., Benhaoua, A., Jlassi, M. & Benhaoua, B. Structural, optical and electrical properties studies of ultrasonically deposited tin oxide (SnO_2_ ) thin films with different substrate temperatures. *Superlattices Microstruct.* **86**, 403-411, (2015).

26 Kompa, A., U, C., Kekuda, D. & Rao K, M. Investigation on structural, optical and electrical properties of Nd doped titania films and application of optical model. *Mater. Sci. Semicond. Process* **121**, (2021).

27 Masouleh, F. F., Sinno, I., Buckley, R. G., Gouws, G. & Moore, C. P. Characterization of conductive Al-doped ZnO thin films for plasmonic applications. *Appl. Phys. A* **124**, (2018).

28 Fernandes, G. E., Lee, D.-J., Kim, J. H., Kim, K.-B. & Xu, J. Infrared and microwave shielding of transparent Al-doped ZnO superlattice grown via atomic layer deposition. *J. Mater. Sci.* **48**, 2536-2542, (2012).

29 Mallick, A., Ghosh, S. & Basak, D. Highly conducting and transparent low-E window films with high figure of merit values based on RF sputtered Al and In co-doped ZnO. *Mater. Sci. Semicond. Process* **119**, (2020).

30 Chen, C.-W. *et al.* Frequency-dependent complex conductivities and dielectric responses of indium tin oxide thin films from the visible to the far-infrared. *IEEE J. Quantum Electron.* **46**, 1746-1754, (2010).

31 Ananthanarayanan, D. *et al.* Mid-infrared characterization and modelling of transparent conductive oxides. *Sol. Energy* **209**, 424-430, (2020).

32 Zhang, L. *et al.* Correlated metals as transparent conductors. *Nat. Mater.* **15**, 204-210, (2016).

33 Boileau, A. *et al.* Tuning of the optical properties of the transparent conducting oxide SrVO_3_ by electronic correlations. *Adv. Opt. Mater.* **7**, (2019).

34 Chuai, Y. *et al.* Highly infrared-transparent and p-type conductive CuSc_1−x_Sn_x_O_2_ thin films and a p-CuScO_2_:Sn/n-ZnO heterojunction fabricated by the polymer-assisted deposition method. *RSC Adv.* **6**, 31726-31731, (2016).

35 Kim, J. Y. *et al.* Infrared conductivity and carrier mobility of large scale graphene on various substrates. *J. Nanosci. Nanotechnol.* **12**, 5816-5819, (2012).

36 Wang, Z., Chen, C., Wu, K., Chong, H. & Ye, H. Transparent conductive oxides and their applications in near infrared plasmonics. *Phys. Status Solidi A* **216**, (2019).

37 Kim, H. *et al.* Effect of film thickness on the properties of indium tin oxide thin films. *Journal of Applied Physics* **88**, 6021-6025, (2000).

38 Kim, H. *et al.* Electrical, optical, and structural properties of indium–tin–oxide thin films for organic light-emitting devices. *Journal of Applied Physics* **86**, 6451-6461, (1999).

39 Abe, Y. & Ishiyama, N. Polycrystalline films of tungsten-doped indium oxide prepared by d.c. magnetron sputtering. *Materials Letters* **61**, 566-569, (2007).

40 Koida, T., Fujiwara, H. & Kondo, M. High-mobility hydrogen-doped In_2_O_3_ transparent conductive oxide for a-Si:H/c-Si heterojunction solar cells. *Solar Energy Materials and Solar Cells* **93**, 851-854, (2009).

41 Hamberg, I. & Granqvist, C. G. Evaporated Sn‐doped In_2_O_3_films: Basic optical properties and applications to energy‐efficient windows. *Journal of Applied Physics* **60**, 123-160, (1986).

42 Wang, Z., Chen, C., Wu, K., Chong, H. & Ye, H. Transparent conductive oxides and their applications in near infrared plasmonics. *physica status solidi (a)* **216**, (2019).

43 Cheng, Y. *et al.* Understanding the structure and properties of sesqui-chalcogenides (i.e., V_2_VI_3_ or Pn_2_Ch_3_ (Pn = pnictogen, Ch = chalcogen) compounds) from a bonding perspective. *Adv Mater* **31**, e1904316, (2019).
